# The evolution of genomic and epigenomic features in two *Pleurotus* fungi

**DOI:** 10.1038/s41598-018-26619-7

**Published:** 2018-05-29

**Authors:** Zhibin Zhang, Jiawei Wen, Juzuo Li, Xintong Ma, Yanan Yu, Xiao Tan, Qi Wang, Bao Liu, Xiaomeng Li, Yu Li, Lei Gong

**Affiliations:** 1 Key Laboratory of Molecular Epigenetics of the Ministry of Education (MOE), Northeast Normal University, Changchun, 130024 China; 2 Jilin Academy of Agricultural Sciences (JAAS), Changchun, 130033 China; 3 Engineering Research Center of the Ministry of Education (MOE) for Edible and Medicinal Fungi, Jilin Agricultural University, Changchun, 130118 Jilin, China

## Abstract

*Pleurotus tuoliensis* (Bailinggu, designated Pt) and *P. eryngii* var. *eryngii* (Xingbaogu, designated Pe) are highly valued edible mushrooms. We report *de novo* assemblies of high-quality genomes for both mushrooms based on PacBio RS II sequencing and annotation of all identified genes. A comparative genomics analysis between Pt and Pe with *P. ostreatus* as an outgroup taxon revealed extensive genomic divergence between the two mushroom genomes primarily due to the rapid gain of taxon-specific genes and disruption of synteny in either taxon. The re-appraised phylogenetic relationship between Pt and Pe at the genome-wide level validates earlier proposals to designate Pt as an independent species. Variation of the identified wood-decay-related gene content can largely explain the variable adaptation and host specificity of the two mushrooms. On the basis of the two assembled genome sequences, methylomes and the regulatory roles of DNA methylation in gene expression were characterized and compared. The genome, methylome and transcriptome data of these two important mushrooms will provide valuable information for advancing our understanding of the evolution of *Pleurotus* and related genera and for facilitating genome- and epigenome-based strategies for mushroom breeding.

## Introduction

The *Pleurotus eryngii* species complex in basidiomycetes, encompassing the largest number of species in the oyster mushroom genus (*Pleurotus*), constitutes many kinds of edible mushrooms such as Bailinggu (*P. tuoliensis*) (Pt), Xingbaogu (*P. eryngii* var. *eryngii*) (Pe), Aweigu (*P. eryngii* var. *ferulae*) and so forth^1–3^. As the two most widely cultivated edible mushrooms, Pe and Pt have been commercialized as nutritional, medicinal, industrial and animal feed products^4–6^. Prior biogeographical studies revealed that Xingbaogu was broadly distributed from France and Spain to western China and some countries in the Mediterranean regions^7^; however, as a native mushroom discovered in western China, wild Bailinggu is primarily distributed in narrow geographical areas where its plant hosts reside^8,9^. Regarding the taxonomy of these two mushrooms, their exact phylogenetic relationship is still debated, although recent phylogenetic analyses support treating the two mushrooms as distinct species^3,10^.

Well known as the white-rot fungi, *Pleurotus* species exhibit wood-decay properties that cause degradation of components of the host plant cell wall (PCW), such as lignin, cellulose, and hemicelluloses^11^. Although belonging to the same genus, host specificity or preference for decayed wood is characterized by the *Pleurotus* taxa: *Pleurotus ostreatus* is often found on dying or dead-standing deciduous broadleaf trees such as beech and oaks^12,13^, whereas Bailinggu and Xingbaogu are associated with a different range of Apiaceae hosts, with the former only associated with *Ferula* spp. and the latter associating with broader hosts such as *Eryngium* spp., *Peucedanum* spp. and *Opopanax chironium*^14^. Additionally, morphology^9,14^ (Figure S1), cultivation conditions^15,16^ and genetic diversity^17^ are distinct between Bailinggu and Xingbaogu. However, the genomic basis underpinning phenotypic variation, adaptation diversity and host specificity among the *Pleurotus eryngii* species complex remains largely unexplored. Although genomes of the two Pleurotus species have been sequenced^18–20^, exploration of these characteristics still requires comprehensive diagnoses of their genomic and epigenomic features, which entails more complete genomic information.

As an important and relatively stable epigenetic modification, DNA methylation is involved in transposable element (TE) silencing, chromatin structure, regulation of gene expression and genetic recombination, which affect various fundamental biological processes and may serve as raw material for major evolutionary innovations^21–23^. Furthermore, recent studies revealed that heritable DNA methylation serves as a molecular link between genotype, environment and phenotype^23,24^. In the fungus kingdom, DNA methylation mainly occurs in TEs, probably as an essential genome defense mechanism to repress their proliferation, but the methylation states of TEs may also affect the expression of neighboring genes^25,26^. Similar to the situation in mammals^27^, DNA methylation in fungi such as basidiomycetes and ascomycetes is mainly in the CG context, which is maintained by the DNA methyltransferase Masc2s of DNMT1^28^. Furthermore, the recently identified fungi-specific DNA methyltransferase Rad8 in basidiomycetes species may function in *de novo* DNA methylation related to the small interference (si)-RNA-directed DNA methylation (RdDM) pathway in plants^28,29^. Nevertheless, prior studies of whole-genome profiles of DNA methylation and siRNA in fungi only focused on a few ascomycetes and basidiomycetes species^30–32^ without involving any *Pleurotus* taxa. Moreover, there are no direct comparisons of DNA methylation profiles between related species in *Pleurotus*.

In this study, we *de novo* assembled the genomes of *P. tuoliensis* and *P. eryngii* var. *eryngii*, both of which belong to the *P. eryngii* species complex. Detailed comparative analyses indicated that the two genomes diverged extensively with synteny of approximately one-third of the protein-coding genes being disrupted since their divergence *ca*. 18 million years ago (MYA) based on phylogenetic analyses. Remarkably, despite the two *Pleurotus* genomes harboring a similar content of TEs and protein-coding genes, the extent of their sequence divergence supports the viewpoint that Pt is a separate species of the *Pleurotus eryngii* species complex. Variation of genes encoding wood-decay-related enzymes explains their variable adaptation and host specificity. Based on genome assemblies, their DNA methylation profiles at the whole-genome level, i.e., methylomes, were characterized and compared with respect to their pattern, establishment and maintenance. Conserved negative regulatory effects of DNA methylation on the expression of genes adjacent to TEs were also explored. Taken together, our two assembled *Pleurotus* genomes and characterized methylomes and transcriptomes offer a collection of valuable genomic, epigenomic and gene expression resources for future research on evolution and functional genomics in *Pleurotus* and related genera as well as on more efficient breeding of improved edible mushroom cultivars.

## Results

### Genome sequences of *P. tuoliensis* and *P. eryngii* var. *eryngii*

**Figure 1:**
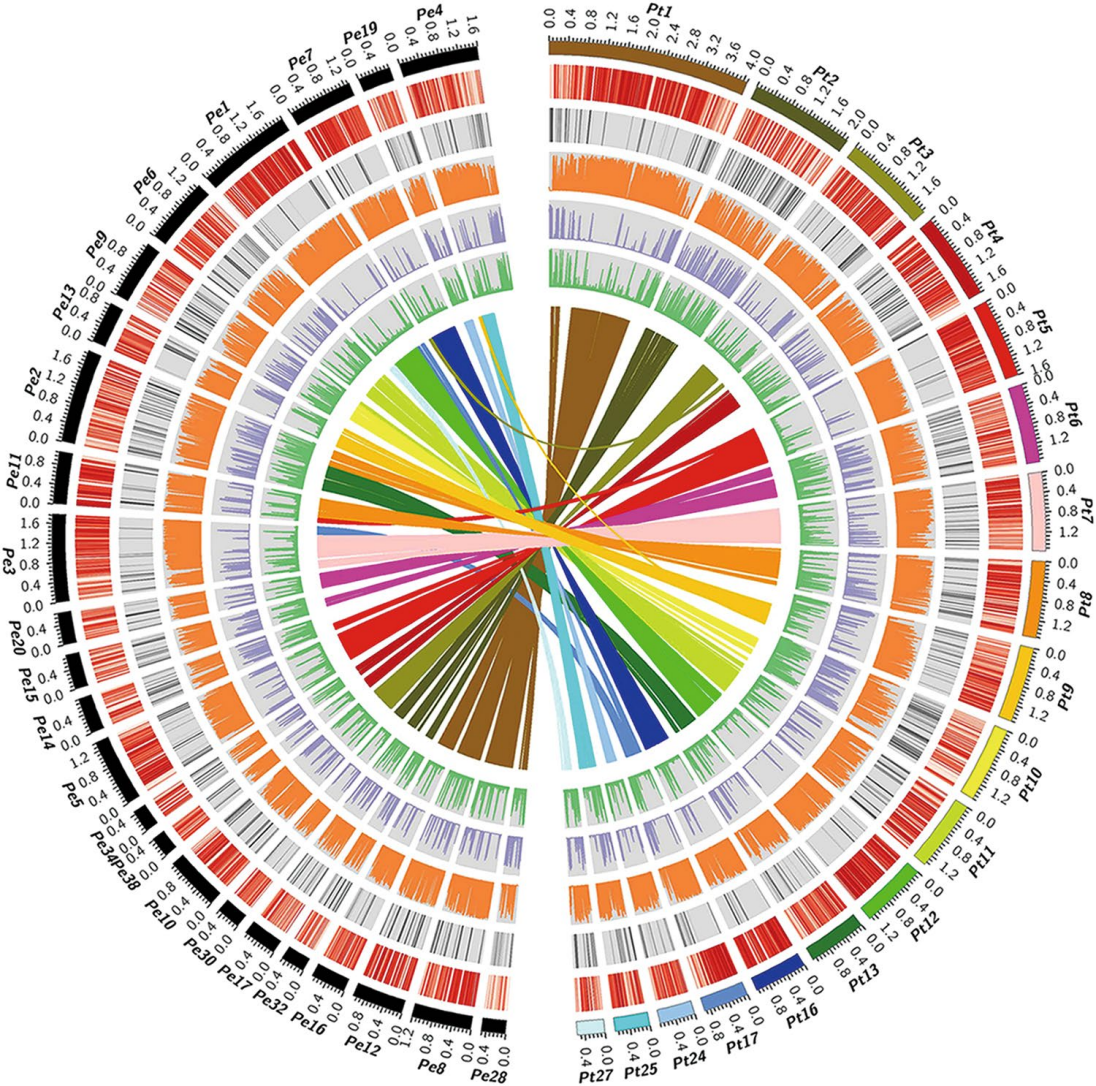
Genomic features of *P. tuoliensis* (Pt) and *P. eryngii* var. *eryngii* (Pe) tabulated in circos plot. The circles from outside to inside represent length of contigs (colored and black stripes represent contigs derived from Pt and Pe, respectively), gene density, repeat elements content, coverage of RNA-seq reads in mycelium, DNA CG methylation level, siRNA reads coverage, respectively. Each feature was calculated based on 10 kb non-overlapping windows. Synteny gene pairs are linked by colored lines in the inner-most circle.

Genomes of Pt and Pe were sequenced on the PacBio RS II platform, which generated 554,694 and 609,010 clean, SMRT long reads with 100- and 125-fold coverage depths (5.6 Gb and 6.3Gb), respectively (Materials and Methods). Draft genome sequences of both strains were independently *de novo* assembled. The draft genome sequences of Pt and Pe contain 106 and 153 contigs and encompass a total length of 48.2 Mb (contig N50 = 1.08 Mb) and 49.9 Mb (contig N50 = 0.55 MB), respectively (Table 1). Relative to the earlier genome sequences of Pt and Pe^18–20^, both our assembled Pt and Pe genome contained fewer contigs but a relatively larger genome size and longer N50 values (Table S1). The deeper sequencing depth by the PacBio RS II platform enabled us to assemble more-complete genomic sequences into longer contigs compared to those produced by Illumina only or Illumina plus shallow PacBio RS II sequencing in the prior studies^18–20^. In total, 13,097 and 13,212 protein-coding genes were predicted in the Pt and Pe genomes, respectively, by running a PASA annotation pipeline, of which 92.5% (Pt) and 93.4% (Pe) were matched against the Non-redundant (Nr) database (Materials and Methods, Tables 1 and S2). The fewer number of annotated gene models in both our Pt and Pe assembly might be attributed to the use of different gene annotation strategies (different annotation tools and/or parameter settings, Table S1). Approximately 20% of both genomes were annotated as transposable elements (TEs) by the RepeatMasker program (www.repeatmasker.org/) (Materials and Methods, Tables 1 and S3). Notably, significantly higher TE contents were annotated in our assemblies (Table S1) relative to those published previously^18–20^, suggesting our longer PacBio reads are clearly more powerful for the assembling of intact TEs. Relative densities of annotated genes, TEs and whole-genome expression levels (evaluated as RNA-seq read coverage) are depicted by a Circos diagram (Fig. 1). It was observed that TEs and other repetitive sequences were interspersed by condensed genic regions which were transcribed at high levels (Fig. 1).

**Table 1:** Genomic features of *P. tuoliensis* (Pt), *P. eryngii* var. *eryngii* (Pe) and *P. ostreatus* (Po).

	Pt	Pe	Po
Number of contigs	106	153	11
Length of the genome assembly (Mb)	48.2	49.9	34.3
Contig N50 (Mb)	1.08	0.55	2.26
GC content (%)	50.01	49.07	50.95
Number of protein-coding genes	13097	13213	12330
Average gene length (bp)	1717	1607	1704
Average coding sequence size (bp)	1186	1069	1246
Average number of exons per gene	6.4	6.4	6
Average exon size (bp)	205	185	217
Average intron size (bp)	73	79	76
Average size of intergenic regions (bp)	1932	2145	1080
TE content (%)	20.0	19.4	9.8
RNAseq reads alignment ratio(%)	91.0	81.1	NA

“NA” denotes that corresponding RNAseq data is not available.

The high quality of our assemblies is supported by the relatively complete and accurate independent gene annotations relative to *P. ostreatus* (designated as Po), which was the first sequenced *Pleurotus* fungus^33^. Specifically, by performing the BUSCO analysis^32^, 96.7% (293 out of 303) and 90.8% (275 out of 303) of the core eukaryotic orthologous genes were annotated in Pt and Pe genomes, respectively, which correspond to 96.7% (293 out of 303) of the same sets of orthologs in *P. ostreatus* (Table S4). By analyzing the general gene structure and other related features of the predicted protein-coding genes (i.e., the average gene length, size, and numbers of exons and introns), we found the genome assemblies of both Pt and Pe to exhibit the same complete level as that in the Po assembly (Table 1). Moreover, 91.0% (Pt) and 81.1% (Pe) of RNA-sequencing reads derived from monokaryotic mycelia were aligned accurately to the corresponding draft genome assembly (Table 1). Consequently, the high quality of both genome assemblies ensures the feasibility and reliability of the following comparative genomic and epigenomic analysis.

### Comparative genomics among *P. tuoliensis, P. eryngii* var. *eryngii*, and *P. ostreatus*

Available genome assemblies of Pt, Pe, and Po facilitate the exploration of their genomic variation from an evolutionary viewpoint. Accordingly, we made pairwise comparisons among assemblies of our sequenced Pt, Pe and Po (which served as the outgroup species) genomic features and specific components.

Regarding the overall genome size, the two assembled genomes of Pt and Pe (48.2 and 49.9 Mb, respectively) are similar and both are larger than that of Po (~34.3 Mb). The higher proportion of TE content, especially the highly abundant long-terminal repeat (LTR) TEs in Pt and Pe (19.96% and 19.36% TEs; 11.74% and 12.35% LTR TEs, respectively) than those in Po (9.80% TEs and 5.76% LTR TEs) may largely explain the overall differences in genome size among the three genomes (Tables 1 and S2). More detailed interrogation of the LTR TE insertion sites suggested that more recent TE insertion events occurred since the divergence of Pt and Pe from their last common ancestor ~18 MYA, as described below; these relatively new insertions have been superimposed on the remnants of more ancient TE insertions that occurred ~ 20 MYA (described below), i.e., before the speciation of Po (Fig. S2). Thus, less abundant LTR TEs could account for the condensed genome in the outgroup Po species. Additionally, the diluted and shorter genes in genomes of the closely related Pt and Pe could contribute to their lower GC content (50.20% and 49.37%, respectively) relative to that in Po (50.95% in Table 1)^34^.

Using the established assembly of Po as a reference, the contigs of both Pt and Pe were successfully mapped to the corresponding chromosomes of Po via reference to their component synteny blocks in Po (Fig. S3). Pairwise synteny comparisons revealed certain proportions of gene pairs in synteny blocks between Po and Pt (64.1% of Pt genes in 81 blocks), Po and Pe (61.3% of Pe genes in 111 blocks), as well as Pt and Pe (61.0% of Pt genes and 61.6% of Pe genes in 132 blocks) (Fig. S4 and Table S5). It is clear that most orthologs in Pt and Pe were maintained in conserved orders since their divergence from Po (Table S5, further detailed below). However, as noted above, there were also many genes lacking synteny in each pairwise comparison (averaged as 37.1% of all genes residing out of the foregoing synteny blocks), which could be the result of genomic reshuffling in the course of independent genome evolution in the respective species.

### Phylogenetic comparisons among *P. tuoliensis, P. eryngii* var. *eryngii*, and *P. ostreatus*

**Figure 2:**
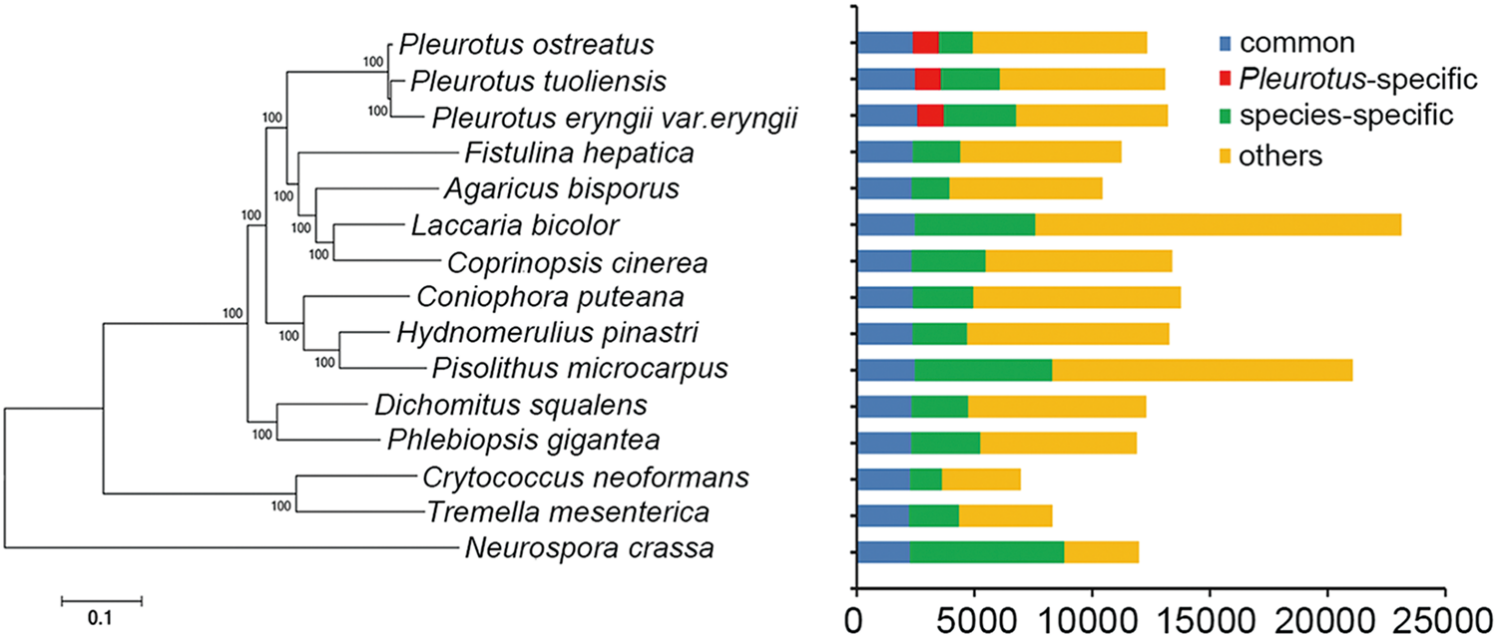
Phylogenetic relationship of conserved single-copy orthologs identified in fifteen fungal species. The maximum likelihood (ML) phylogenetic tree was constructed from 1385 conserved (existing in all analyzed fungal species) single-copy orthologs (aligned by MAFFT) using FastTree with the model of LG + CAT. In addition, those common conserved (including both conserved single-copy and multiple-copies orthologs), *Pleurotus*-specific (existing in all three *Pleurotus* taxa whereas absent in other fungi), taxa-specific (uniquely existing in one specific *Pleurotus* genome), and other types of orthologs are categorized and tabulated, respectively.

Based on limited molecular markers and genes, recent phylogenetic studies suggested that Pt should be an independent species^3,10^. To further confirm the phylogenetic/taxonomic position of Pt from a genome-wide perspective, we constructed a highly integrative consensus species tree based on whole-genome orthologs identified in Pt, Pe, and Po. Specifically, by inputting all the annotated genes identified in these three *Pleurotus* genomes and an additional 12 sequenced representative fungal species into OrthoMCL^35^, we identified featured orthologs in the three *Pleurotus* genomes, which included 2,216–2,573 highly conserved orthologs existing in all analyzed fungal species, 1,090–1,124 orthologs existing in the three *Pleurotus* genomes but absent in any other fungus genome, and 2,499, 3,062 and 1,438 genome-specific genes identified in Pt, Pe, and Po, respectively (Fig. 2). Intriguingly, the *Pleurotus*-specific genes were found to be enriched in gene ontology (GO) terms of protein phosphorylation and protein kinase activity in both Pt and Pe (Table S6). Notably, the taxa-specific genes derived from both Pt and Pe were included in identical GO terms that are mainly involved in oxidation-reduction processes, transmembrane transport and carbohydrate metabolic processes, indicating that the two *Pleurotus* taxa may possess divergent enzymes related to the aforementioned specific biological processes.

The 1,385 single-copy, highly conserved orthologs identified in all these fungus taxa were subjected to the construction of a consensus maximum likelihood (ML) phylogenetic tree (Materials and Methods). It was found that Pt and Pe clustered into one clade with a sister branch linking the tip of Po (Fig. 2). Of note, the internal genetic distance between Po and Pt was shorter than that between Po and Pe (Fig. 2). Neutral molecular clock calculations estimated that the divergent times between Po and clades of Pt and Pe were approximately 20 MYA and between Pt and Pe were approximately 18 MYA (Materials and Methods). Such ancient divergence between Pt and Po implicates their sequence divergence in separation, which is not observed between the Pe varieties^3,14^. Our results thus support the recent suggestion for allocating Pt to a higher taxonomical position, i.e., as a separate species (*P. tuoliensis*) rather than classifying it as a variety or subspecies^3,14^. Finally, the observed shortest ML distance and least divergent time between Pt and Pe is in line with previous results showing that taxa in the *P. eryngii* complex (including Pt and Pe) possess the closest phylogenetic relationship^14^.

### Characterization and comparison of wood-decay-related genes in *P. tuoliensis*, *P. eryngii* var. *eryngii*, and *P. ostreatus*

Known as the white-rot fungi, the *Pleurotus* species have evolved a wood-decaying capacity, i.e., the capacity to degrade components of the host plant cell wall (PCW)^36^. Po prefers to grow on rotten deciduous broadleaf trees^12,13^. Relative to the *Pleurotus* *eryngii* complex (including Pe) growing on roots and lower stem residues of Apiaceae (umbellifers) plants, Pt has a narrower host range and only grows on *Ferula* species^14^. These characteristics suggest that there are complicated mechanisms underpinning divergent host niches among Pt, Pe, and Po. To understand the genomic basis conferring these *Pleurotus* species’s efficient PCW-decay capacity and their host selection preference, we interrogated the profiles of genes involved in wood degradation such as carbohydrate-acting enzymes (CAZymes) and lignolytic oxidoreductases^37^. By consulting the orthologs of these genes in other related fungi with sequenced genomes, the evolutionary histories of these genes were inferred as detailed below.

**Figure 3:**
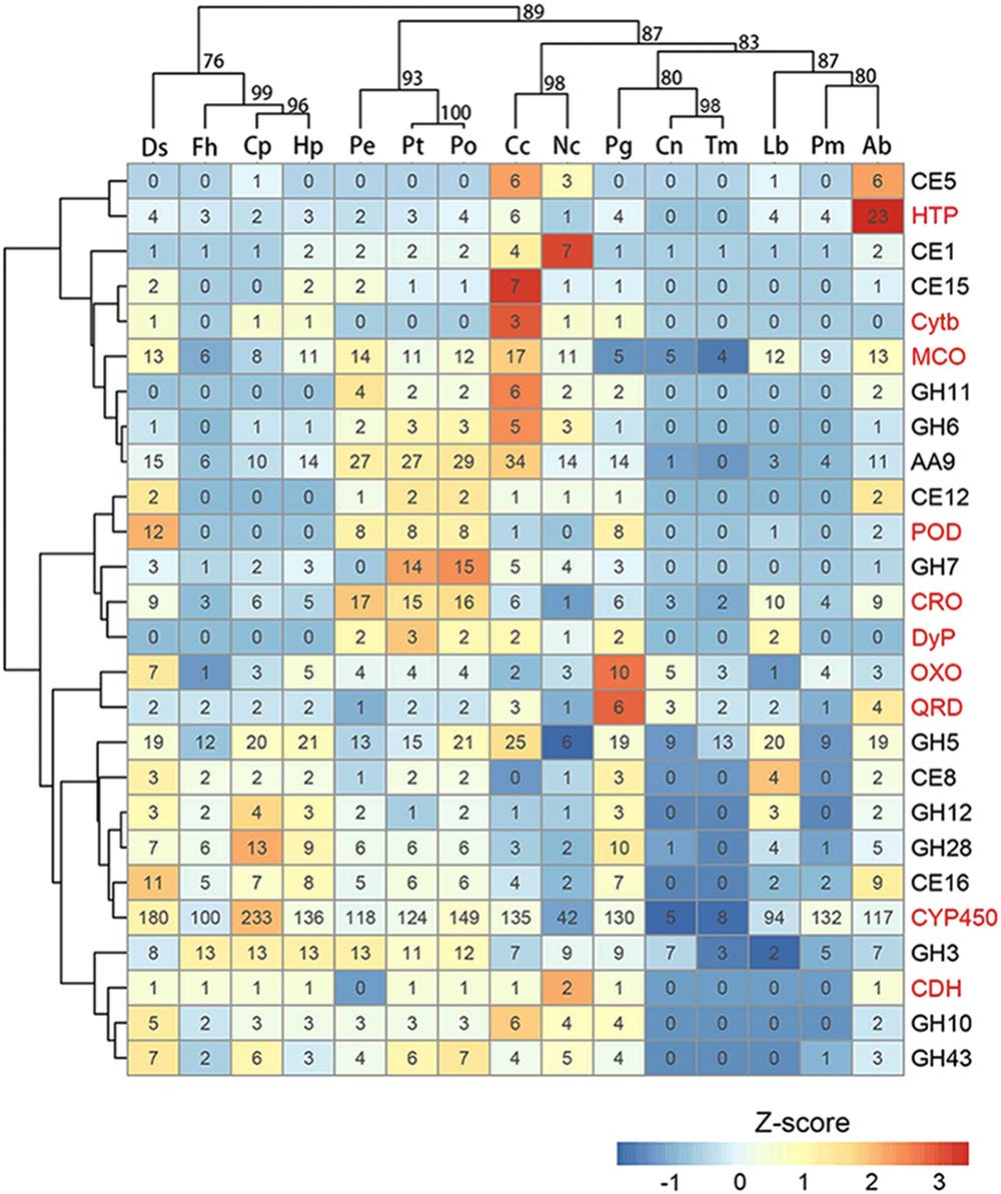
Clustered gene contents of CAZyme and oxidoreductase families in fifteen representative fungi genomes. Hierarchical clustering of wood-decay related genes were completed using the R package pvclust, in which the unbiased (AU) p-values (%) are computed by 1000 bootstrap re-samplings. Respective gene numbers of each gene family in corresponding genome is shown in each cell. Over-represented and under-represented gene families are depicted in red and blue backgrounds, respectively. Within the gene family on the right column, abbreviated names of CAZyme and lignolytic oxidoreductase families are denoted in black and red, respectively. Abbreviations of gene family names: GH, glycoside hydrolases; CE, carbohydrate esterases; POD, class II peroxidases; MCO, multicopper oxidases; CRO, copper-radical oxidases; CDH, cellobiose dehydrogenase; Cytb562, cytochrome b562; OXO, oxalate oxidase/decarboxylases; QRD, quinone reductases; DyP, dye-decolorizing peroxidases; HTP, heme-thiolate peroxidases; P450, cytochromes P450. Abbreviations of species names: Po, *Pleurotus ostreatus*; Pt, *Pleutotus tuoliensis*; Pe, *Pleurotus eryngii* var. *eryngii*; Fh, *Fistulina hepatica*; Ab, *Agaricus* *bisporus*; Lb, *Laccaria bicolor*; Cc, *Coprinopsis cinerea*; Cp, *Coniophora puteana*; Hp, *Hydnomerulius pinastri*; Pm, *Pisolithus microcarpus*; Ds, *Dichomitus squalens*; Pg, *Phlebiopsis gigantean*; Cn, *Cryptococcus neoformans*; Tm, *Tremella mesenterica*; Nc, *Neurospora crassa*.

CAZymes include an important set of enzymes in fungi that are involved in hydrolysis and formation of complex carbohydrates^38,39^. We identified an average number of 330 CAZyme genes in our assembled *Pleurotus* genomes (Table S7). We carried out a phylogenetic analysis focusing on gene family size maintenance and variation of representative CAZyme families in Pt, Pe, and Po to trace their evolutionary histories (Fig. 3 and Table S8). We made the following major observations: First, as core AA (Auxiliary Activities) gene families, AA9 (copper-dependent lytic polysaccharide mono-oxygenases), involving the oxidation of polysaccharides, account for substantial numbers of component genes among our *Pleurotus* genomes compared to those in the corresponding family of the other fungi (Fig. 3). This observation suggests that the AA9 gene family of the three *Pleurotus* genomes might have undergone multiple ancient gene family expansions prior to their speciation from the latest common ancestor and were then stably inherited as orthologs in the respective descendant genomes, whereas the gene family in the other fungal species maintained relative constant smaller sizes throughout their evolutionary histories. Second, with respect to the GH (glycoside hydrolases) gene family, the unique absence of GH7 (known as cellobiohydrolases) in Pe but significantly larger family sizes in Pt and Po may implicate gene family expansion before divergence of the three *Pleurotus* species and specific loss in the Pe genome.

Some key oxidoreductases such as peroxidases (PODs), multicopper oxidases (MCOs) and heme-thiolate peroxidases (HTPs) are sets of enzymes known to confer on fungi the capacity to degrade lignin in their typical host processes^37,40^. A similar comparative analysis as above parsed these gene families into the same subsets of categories in terms of their family-size evolutionary features (Fig. 3 and Table S8): (*i*) the Class II lignolytic PODs, including lignin, manganese, and versatile peroxidases (LiP, MnP, and VP), were markedly rich in the three *Pleurotus* genomes as well as other white-rot species including *Phlebiopsis gigantean* and *Dichomitus squalens*, which was in agreement with previous results showing that this kind of peroxidase that is involved in lignin degradation is enriched in white-rot fungal species^41,42^; (*ii*) the CRO family (copper-radical oxidases)^43^, containing Glyoxal oxidase (GLX) and other CROs, was relatively abundant in Pt, Pe, and Po. CROs, especially GLXs, produce peroxide to support peroxidase activity for lignin degradation, and the relatively enriched CROs among the three *Pleutotus* taxa indicate that their high-capacity for lignin degradation is likely the consequence of adaptation to host cell wall composition and different environmental conditions compared with other fungal species.

Certain CAZymes and lignolytic oxidoreductase gene families, such as GH5 (21 genes in Po vs. 15 and 13 genes in Pt and Pe) and CYP450 (149 genes in Po vs. 124 and 120 genes in Pt and Pe), have expanded in Po relative to Pt and Pe, and those families with relative fewer component genes in Pt than in Pe, such as GH11 (2 genes in Pt vs. 4 genes in Pe), could have played important roles in divergent cell wall degradation, which eventually results in discrepancies in host preference (Fig. 3).

The proportion of different types of wood-decay-related genes is similar between assemblies of strains 183^19^ and our Pe (Fig. S5). However, we note that the total number of annotated CAZyme-coding genes in our assembly is less than that annotated in strain 183, whereas oxido-reductases are greater. These differences might be attributed to using different gene prediction tools with different parameter settings.

### Comparison of *P. tuoliensis* and *P. eryngii* var. *eryngii* methylomes

The availability of well-assembled genomes of Pt and Pe allowed us to analyze their DNA methylation modifications at a genome-wide scale in detail. Given the crucial role of DNA methylation in regulating gene expression and maintaining genome stability in eukaryotes, we generated whole-genome DNA methylation profiles in Pt and Pe and conducted a comparative analysis.

**Figure 4:**
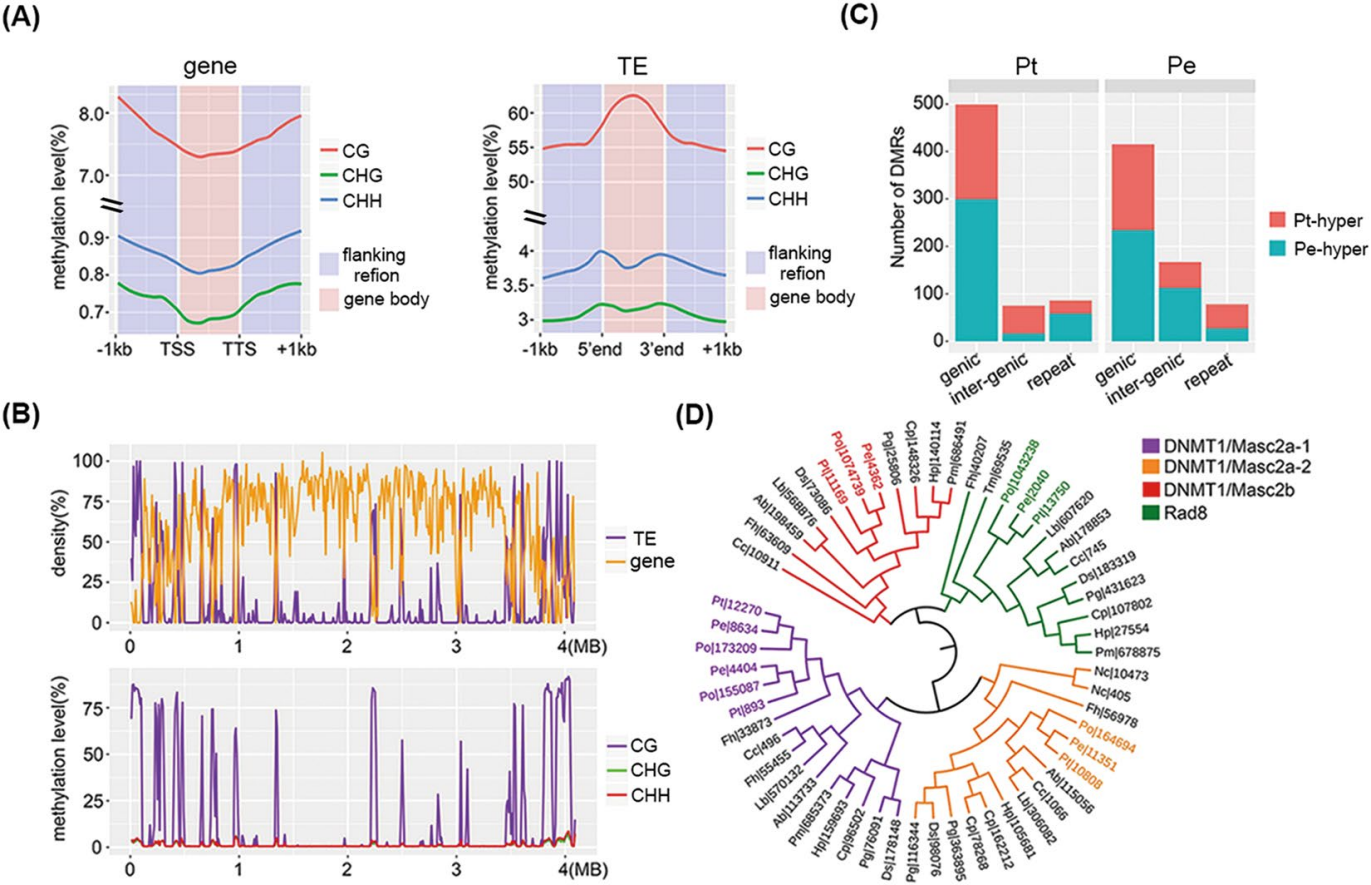
Establishment of DNA methylation profiles in *P. tuoliensis* (Pt) and *Pleutotus eryngii* var*. eryngii* (Pe). (**A**) Averaged DNA methylation levels within (enclosed between TSS and TTS) and around (flanking +1kb) genic and TE regions in Pt; (**B**) Density curves of genes and TEs (upper) and distribution of DNA methylation levels (in the contexts of CG, CHG and CHH, bottom) along the contig 1 of Pt genome; (**C**) Numbers of identified DMRs (hyper- and hypo- methylated regions) within genic (defined as regions of gene body accompanied with flanking +1kb interval), inter-genic, and repeat regions of Pt and Pe; (**D**) Phylogenetic tree of gene homologs encoding DNA methyltransferases in fifteen fungal species. The Maximum Likelihood (ML) phylogenetic tree was constructed based on the DNA methylase domain (PF00145) of 56 DNA methyltransferases. Sequence alignment, tree construction, and further validation were completed using MAFFT and FastTree (with 1000 bootstrapping replicates), respectively.

We first obtained valid methylation sequence data covering more than 87% of cytosine sites in the two genomes (86.7% and 88.6% for Pt and Pe, respectively). Overall, similarly featured DNA methylation profiles were found in Pt and Pe, which was reflected by the following observations: (*i*) preferential ^m^C (methylated cytosine) in a CG context showed significantly higher average methylation levels (14.6% and 16.4% for Pt and Pe, respectively) than those in CHG and CHH contexts (1.4% and 1.7% for CHG and CHH in Pt; 1.3% and 1.5% for CHG and CHH in Pe), which is similar to other fungi^30^; (*ii*) distinct peaks in the curves of DNA methylation levels of TE and genic regions in both species indicate that ^m^CG is mainly located within TEs but is almost depleted in genes (Fig. 4A and Fig. S6A); (*iii*) the two genomes harbor similar genome-wide co-localization patterns of ^m^CG levels and TE densities (Fig. 4B and Fig. S6B); (*iv*) ^m^CG levels in both genomes followed bimodal distributions in which ^m^CGs always clustered at either the lowest (<10%) or highest (>80%) methylation levels (Fig. S7), similar to those reported in plants and other fungi^26,44^. Together, these results indicate that DNA methylation mainly occurred in a CG context and that they may play a major role in silencing TE activity, regulating gene expression, and maintaining genome stability in both Pt and Pe.

Notwithstanding similarities that seem to prevail between Pt and Pe in terms of genomic features as described in the foregoing sections, regional epigenomic divergence in the form of cytosine methylation between Pt and Pe could underpin the developmental divergence in their mycelia and subsequent fruiting stages^17^. Accordingly, we performed detailed comparisons of DNA methylation within the syntenic regions in Pt and Pe and identified 667 DMRs (Differentially Methylated Regions) in Pt and Pe at the mycelium stage (Fig. 4C). We made the following observations: (*i*) Only 1.7% of the syntenic regions were included in the identified DMRs. (*ii*) Genic DMRs (DMRs in gene bodies and their flanking 1 kb regions) accounted for the highest proportion of DMRs in both Pt and Pe (56.2% in Pe and 64.3% in Pt, respectively). (*iii*) There was no significant bias towards either genic Pt- or Pe-hypermethylated DMRs in either species (Fisher exact test, p > 0.05). Therefore, the distinct though limited divergence of DNA methylation in Pt and Pe mainly occurred in their genic regions with no bias to either species. Intriguingly, we note that those genes involved in the genic DMRs of both species (291 and 262 genes in Pt and Pe, respectively) shared common GO terms such as oxidation-reduction process, protein phosphorylation, transmembrane transport and so forth (Table S9). Therefore, genes with DMRs in the two species provide candidate targets for future functional studies on the potential epigenetic basis underlying the morphological and physiological differences between the two *Pleurotus* taxa.

### Possible genetic basis for establishment and maintenance of DNA methylation in *P. tuoliensis* and *P. eryngii* var. *eryngii*

DNA methyl-transferase (DNMTase) and non-coding small interference (si)-RNAs are common essential components in pathways that define and maintain the dynamic DNA methylation levels and patterns in both animals and plants^27^. To explore the possible molecular basis establishing and maintaining the DNA methylation profiles in Pt and Pe, we characterized the composition and potential function of DNMTase-encoding genes in establishment and maintenance of DNA methylation by retrieving their orthology and evolutionary history in the phylogenetic trees and analyzed the potential role of siRNA-directed DNA methylation in these two *Pleurotus* taxa.

Initially, a search for genes harboring the core DNA methyltransferase domain (PF00145) in the proteome of Pt and Pe and a subsequent orthology comparison identified four ortholog groups reported in previous studies on DNMTase in Po (Fig. 4D, DNMT1/Masc2), which included representative fungal DNMTases of DNMT1/Masc2, DNMT2, and Rad8^28^. Our RNA sequence data support the transcriptional expression of all these identified DNMTase genes in monokaryotic mycelia of Pt and Pe (Fig. S8). As illustrated in the phylogenetic tree (Fig. 4D), aside from two ortholog lineages of DNMT1/Masc2 (named DNMT1/Masc2a-1 and DNMT1/Masc2a-2), there was only one ortholog lineage of DNMT1/Masc2b, DNMT2 and Rad8 in all sequenced *Pleurotus* genomes. This observation implies the following: (*i*) the conservative lineage of a single copy of DNMT1/Masc2b, DNMT2, and Rad8, was maintained in each of the three *Pleurotus* taxa without any gene duplication involved, and (*ii*) an ancient duplication of DNMT1/Masc2a gene could have taken place in the common ancestor of the three *Pleurotus* species or even prior to their divergence, and these two DNMT1/Masc2a-1 and DNMT1/Masc2a-2 gene lineages were then stably inherited. Considering the high sequence similarity between DNMT1/Masc2a with mammalian DNMT1, which plays key roles in maintaining ^m^CG^28,45^, such expansion of the DNMT1a family in the three *Pleurotus* genomes might be responsible for maintaining ^m^CG in the three *Pleurotus* genomes. A conserved function in all kingdoms of DNMT2 is for tRNA methylation^30,46^, but its role in fungi remains unclear. Finally, for Rad8, because it contains the characteristic methyltransferase Snf2_N and Helicase_C domains of the Snf2 superfamily^47^, we suspect that it may have important functions in RdDM for achieving *de novo* DNA methylation in fungi.

**Figure 5:**
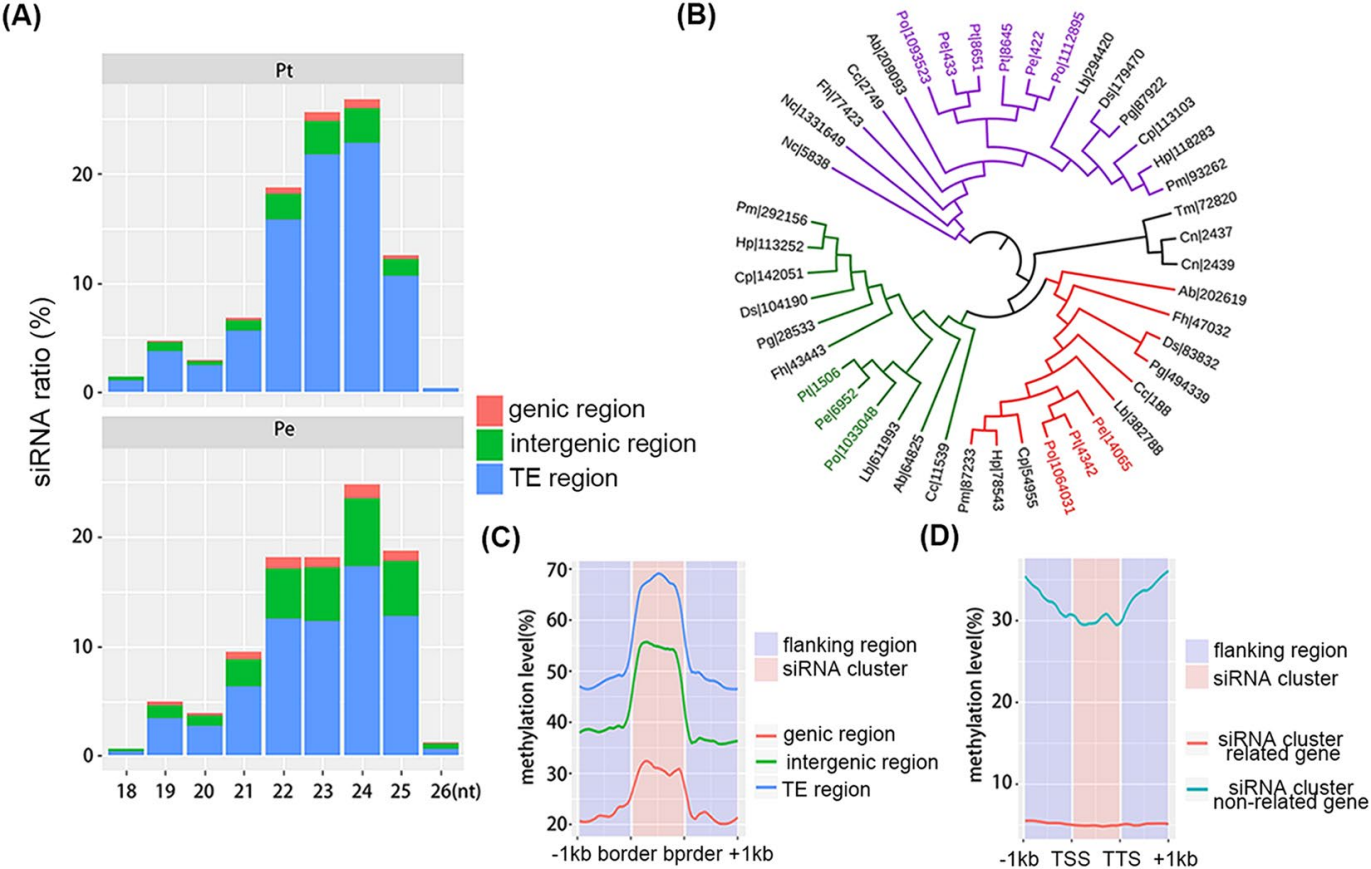
Original and distribution of siRNAs and DNA methylation profiling of siRNA clusters in *P. tuoliensis* (Pt) and *Pleutotus eryngii* var*. eryngii* (Pe). (**A**) Length and genomic region distribution of siRNAs in Pt and Pe are tabulated in bar columns. Genic-regions defined as regions of gene body accompanied with flanking +1kb; (**B**) The Maximum Likelihood (ML) phylogenetic tree of identified *Pleurotus* DCL genes was constructed as described in Fig. 4 based on RNA dependent RNA polymerase domain (PF05183) and different colors denotes diverged clades of *Pleurotus* DCL genes; (**C**) DNA CG methylation level of siRNA clusters located in genic-, TE-, and intergenic-regions, respectively, in Pt; (**D**) DNA CG methylation level in siRNA cluster-related and siRNA cluster-non-related genic contexts in Pt.

We further investigated the possible siRNA-directed RdDM in the two *Pleurotus* genomes by integrated comparative analysis, which included comparing the siRNA content in Pt and Pe, characterizing the functional machinery of siRNA biogenesis and silencing in Pt, Pe, and Po, as well as allocating genome-wide distributions of siRNAs and DNA methylation in Pt and Pe. The following major results were obtained: (*i*) Cleaned siRNAs (after removing noisy RNAs, see Materials and methods) from Pt and Pe showed similar distributions of abundance in terms of sequence length (Pearson's Chi-squared test, p = 0.2303, Fig. 5A), in which 21–25 nt siRNAs were most abundant and the vast majority of siRNAs (>65%) were mapped to repeat regions. (*ii*) Genes encoding the machineries of siRNA biogenesis and silencing, including RdRP (RNA dependent RNA Polymerase), DCL (Dicer-like) and Argonaute proteins were identified and compared among Basidiomycota fungi (except for *Neurospora crassa* belonging to Ascomycota). A relatively conserved composition of siRNA-related genes was observed among Pt, Pe, and Po; however, a discrepancy in the number of DCL (between the two *Pleurotus* species and other fungal species) and RdRP genes (among Pt, Pe, and Po) suggested that the complicated gene duplications occurred before divergence of Pt, Pe, and Po and after Pt’s speciation (Figs 5B, S9 and Table S10). (*iii*) Allocating genome-wide distributions of siRNAs and DNA methylation revealed co-localization of their abundance peaks (Fig. 1). After grouping overlapping or neighboring siRNAs (with distance < 100 bp), we found that 81.6% (1,977 out of 2,424) and 79.3% (2,653 out of 3,347) of siRNA clusters overlapped with 89.5% (2,324 out of 2,597) and 88.3% (2,947 out of 3,338) of known TE components in Pt and Pe, respectively. High ^m^CG levels of TE-related siRNA clusters were also found in both Pt and Pe (Fig. 5A and Fig S10A). Furthermore, genic regions co-localizing with siRNA clusters were found to be highly methylated compared with other genic regions (Fig. 5A for Pt and Fig. S10B for Pe; Mann-Whitney-Wilcoxon test, p <2.2e-16 for both Pt and Pe). These results suggest that siRNA-directed DNA methylation could be an important mechanism in establishing and maintaining the methylome profiles in Pt and Pe via targeting both TEs and genic regions.

### Negative regulation of gene expression by DNA methylation in genic and neighboring TE regions

**Figure 6:**
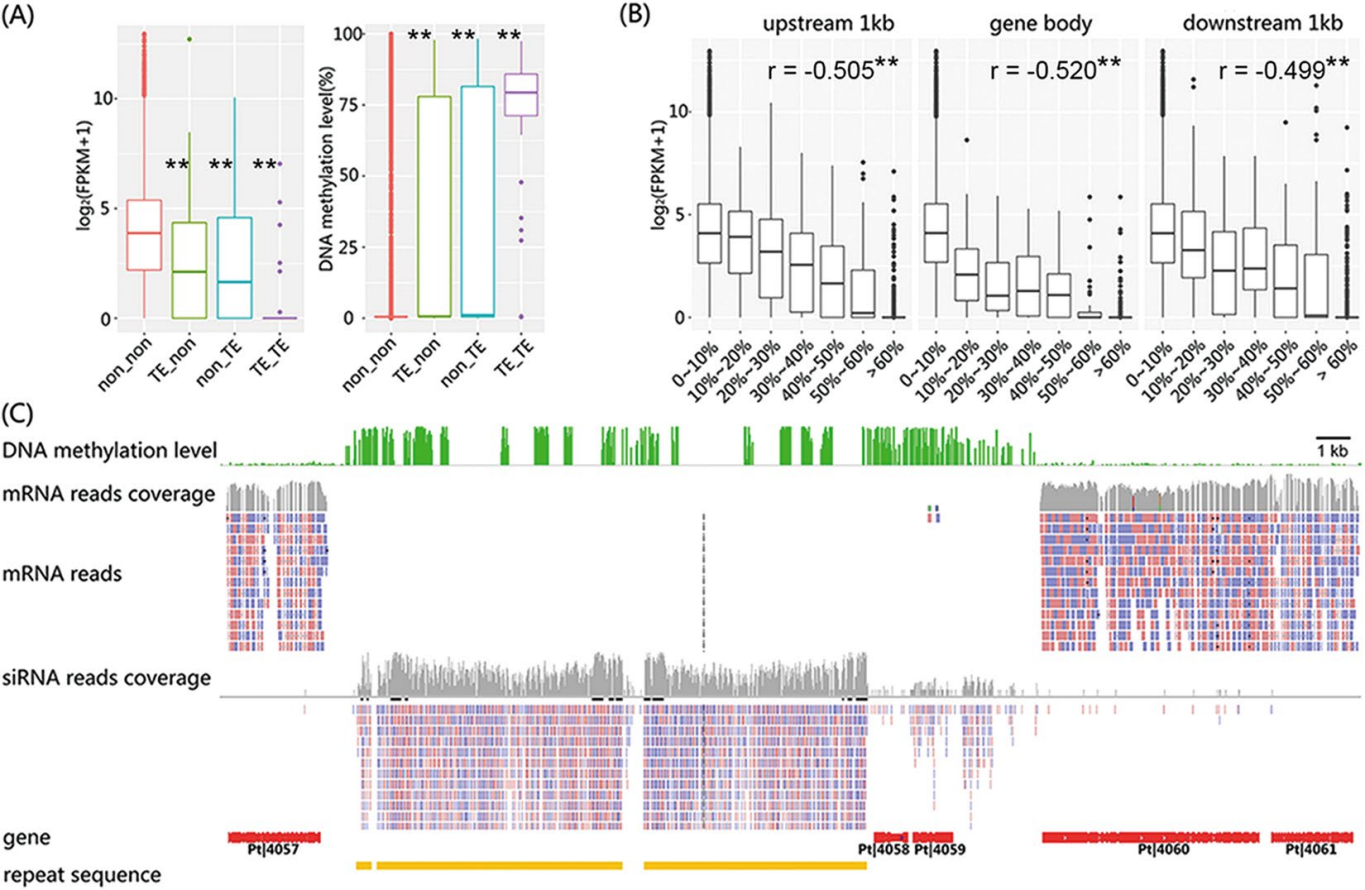
DNA methylation around TE regions negatively regulate expression of adjacent genes in *P. tuoliensis* (Pt). (**A**) Negative impacts of DNA methylation in/around TEs (on the right) on the expression of their neighboring genes (evaluated as FPKM values, on the left) in Pt. “TE_TE”, “non_TE”, “TE_non”, and “non_non” denotes genes flanked with TEs on both upstream and downstream sides, only flanked with downstream TEs, only flanked with upstream TEs, and without any flanking TEs, respectively. Asterisks indicates both levels of gene expression and gene body DNA methylation were significantly different between gene set without any flanking TEs and other gene sets (p < 0.01; Mann-Whitney-Wilcoxon test); (**B**) Significant negative correlation between gene expression and DNA methylation level within corresponding genic regions. On the x axis, genes are categorized into bins of hierarchical DNA methylation levels. Asterisks indicates significantly negative correlation between DNA methylation level in different contexts and gene expression level (p < 0.01; Pearson’s product-moment correlation); (**C**) Exemplary overviewed IGV tracks illustrate the relative distributions of DNA methylation level, mRNA reads abundance, siRNA reads abundance along the ~35 kb region in contig 1: 1,825,947–1,860,003 of Pt.

It is well established that DNA methylation in genic regions per se and adjacent TE regions are capable of regulating their expression at the transcriptional level in both plants and animals^48,49^. A previous study in Po also demonstrated that gene expression was significantly repressed under the influence of more heavily methylated TEs^50^. Inspired by these phenomena, we also investigated the effects of both genic and TE DNA methylation on expression in Pt and Pe. Specifically, a negative correlation of hierarchically attenuated expression with augmented TE methylation was observed for those genes with different numbers of flanking TEs (1 kb-upstream and -downstream from the gene; Mann-Whitney-Wilcoxon test, p<2.2e-16 for both Pe and Pt), which was reflected as expression of genes with no flanking TEs (No TE-gene-No TE) > that of genes with flanking TE at one side (upstream TE-gene or gene-downstream TE) > that of genes with two-sides flanking TEs (upstream TE-gene-downstream TE), whereas there was a reverse relationship in the magnitude of flanking TE methylation in the corresponding categories (Fig. 6A and Fig. S11A). These results showed that TEs silence the expression of their neighboring genes by means of DNA methylation. For genic regions, significant negative correlations between DNA methylation and corresponding gene expression in 1 kb-upstream, 1 kb-downstream and gene body regions were also detected in Pt and Pe (Fig.  6B and Fig. S11B for Pt and Pe, respectively). This is consistent with a previous study of the plant pathogen *Magnaporthe oryzae*^32^, which showed that DNA methylation is associated with transcript abundance of genes in a context-dependent manner.

## Discussion

*Pleurotus* species, a widely commercialized basidiomycete with high nutrient and medicinal values, accounts for approximately one-fourth of the worldwide annual production of edible mushrooms^2^. Focusing on representative species within *Pleurotus*, we completed draft genome assemblies of *P. tuoliensis* and *P. eryngii* var. *eryngii*, annotated their protein-coding genes and TEs, made within-genus comparative genomic analyses with an outgroup *Pleurotus ostreatus*, and explored the potential genomic mechanisms of their distinct habitat adaptions. Supported by genome assemblies, profiles of DNA methylomes of Pt and Pe were characterized in detail and potential factors for establishing and maintaining the DNA methylation profiles were explored. Therefore, our study provides an important genomic and epigenomic resource for understanding the pattern and mechanisms of genetic and epigenetic divergence within two *Pleurotus* species, which will have implications for future genome-based improvements of *Pleurotus* mushrooms.

### The two gene-condensed *Pleurotus* genomes underwent heterogeneous transposable element dynamics that contributed to within-genus genome size differences

It is well-established that fungal species harbor small genomes with highly specific genic regions and reduced non-coding regions^25,41^. In line with this, the sequenced genomes of *Pleurotus* taxa were assembled to less than 50 M bp but exhibited large numbers of annotated protein-coding genes and few constituent TEs (Fig. 1 and Table 1). However, as aforementioned, there were genome size variations among the three *Pleurotus* taxa, which could have arisen from unequal TE bursts and retention and genic region variations (Fig. S2). Accordingly, considering that specific environmental conditions could cause TE activation^51^, adaptations to the cold habitats of high altitude and latitude of Pt and Pe could have induced their recent independent TE bursts, which were absent in Po that are distributed in temperate climate regions^14,52^. Similarly, preferential retention of ancient TEs in Pt and Pe could also be associated with natural selection for their roles in regulating the expression of neighboring genes in their respective genomes. The genic structural variation that was revealed as longer exonic regions in Po (Table 1) might be due to more complicated gene regulation in this species, which is probably necessary for Po to decay a wider range of hosts^53^.

### Rapid gain of taxon-specific genes and reshuffling and/or loss of gene orthologs in the two *Pleurotus* species

Comparisons of genic sequence similarity and gene orders between taxa of a given genus facilitates tracing of the genic homology and synteny within a given evolutionary time-frame^54,55^. In our study, considering the estimated divergence time between Pt and Pe (~18 MYA), the much greater number of taxa-specific genes (~2,500 to ~3,100) than that in the outgroup Po (~1,400) indicates a rapid gain of novel genes in the independent evolutionary trajectories of Pt and Pe compared to that of the outgroup Po (Fig. 2). Given the significantly greater TE content in both Pt and Pe than in Po, as described above, a possible scenario to explain the extra genes in both Pt and Pe is that more TEs, and hence more frequent TE-mediated recombinations, have generated more gene duplications in Pt and Pe, which could have become novel genes after subsequent neo-functionalization^56^. Additionally, relative to the well-maintained synteny among closely related higher multicellular species of similar divergence time^57,58^, a loss of ~37% synteny genes between Pt and Pe suggests a rapid reshuffling and/or loss of gene orthologs within each taxa.

### Taxonomical position of *P. tuoliensis* is verified by a consensus phylogenetic gene tree based on genome-wide sampling

The exact taxonomic status of Pt has remained controversial since its first collection by Mou^59^. Previous taxonomic studies with *Pleurotus* were mainly based on phylogenetic comparisons of morphological characteristics and limited gene loci (such as the ITS sequence)^3,8,9,14^. Considering the apparent limitations such as incomplete lineage sorting and character convergent evolution^60,61^, the taxonomical status of Pt cannot be unequivocally determined by these existing studies. The availability of improved genome sequences enables construction of a consensus phylogenetic gene tree by integrating whole-genome single-copy gene orthologs in Pt and related taxa, which offers an unprecedented opportunity to resolve this issue (Fig. 2). Our results indeed confirm that Pt should be placed at a higher taxonomical status, i.e., as an independent species. Considering the recent genetic population structure analysis showing low gene introgression with *P. eryngii* var. *eryngii* and *P. eryngii* var. *ferulae*^17^ and narrower host specificity^14^, it is reasonable to assume that the 18 MY geographical isolation of Pt and difference in biotope could have led to speciation from the other taxa of *Pleurotus*.

### Evolution of wood-decay-related genes uncovers potential candidate genes conferring adaptation and host specificity in the *Pleurotus* taxa

A focal issue in comparative genomics of basidiomycetes concerns the wood-decay fungi^37^. To gain further understanding of the wood-decay characteristics of the *Pleurotus* taxa, characterizing the composition and tracing the evolutionary history of their wood-decay-related genes at a whole-genome level are expected to provide new insights into their adaption and host specificity. In our study, three *Pleurotus* taxa were determined to harbor full sets of genes coding for carbohydrate- and lignin-active enzymes (Fig. 3), which may constitute their genetic underpinnings as white-rot wood-decay fungi^33^. Clustering of these enzyme-coding genes in terms of their copy abundance implies an overall similar nutritional mode for these three *Pleurotus* taxa (Fig. 3). In particular, for those ancient gene copies encoding the CAZymic and lignolytic enzymes, their conserved composition could be associated with the adaptation/colonization of *Pleurotus* taxa to their respective ecological niches; however, additional evidence from further gene expression analysis is needed. Host specialization is common in basidiomycetes, among which the *P. eryngii* species complex has developed a certain degree of host specificity^3^. The wood-decay genes with discrepant copy numbers uncovered in the three taxa that we studied could be promising candidates for determining their respective host specificities. Additional decay assays of each taxon in wood substrates coupled with temporal-spatial expression analysis of these genes in the decay process may further characterize the gene(s) involved in host specialization of *Pleurotus* taxa.

### DNA methylation in TE regions of two *Pleurotus* species established via RdDM and maintained by DNMT1/Masc2a negatively regulates gene expression

It is known that both genetic and epigenetic variations underlie evolutionarily relevant and useful phenotypes. However, to our knowledge, genome-scale DNA methylation divergence between closely related fungal taxa remains unexplored. Given the general genomic similarity between Pt and Pe in *Pleurotus*, a comparative analysis of methylomes between these two taxa may provide novel insights into the molecular basis of their phenotypic differences.

Consistent with studies in other single fungal species^25,26,30^, we found that methylation at CG sites (^m^CG) in TE regions of Pt and Pe is the major pattern of DNA methylation (Fig. 4). To obtain clues regarding the molecular mechanisms for the establishment of DNA methylation in the studied *Pleurotus* taxa, our identification of the full sets of genes encoding the enzymes in the siRNA-biogenesis machinery and co-localization of siRNA abundance and TE DNA methylation profiles support the possibility that the observed TE DNA methylation is likely established by the RdDM pathway (Fig. 4 and Fig. 5), which is known to ubiquitously exist in the plant kingdom. However, since there was no DNMT3 ortholog identified in the studied *Pleurotus* fungi, we suspect that these fungi probably employ Rad8 as an integrant of the RdDM machinery rather than involving any orthologs of DNMT3, given that Rad8 contains both the DNA methyltransferase required by RdDM and the SNf2 domains^28^. Since the overall DNA methylation in the two *Pleurotus* fungi is ^m^CG, as in mammals, Rad8 probably maintains DNMT3’s functional specificity, albeit with the occurrence of domain reorganization events. Regarding the maintenance of DNA methylation, the mammalian DNMT1 ortholog, DNMT1/Masc2, was found to be highly similar between mammals and fungi both in protein structure and possible biological functions^28^. Accordingly, DNMT1/Masc2 is also likely responsible for maintaining DNA methylation (predominantly ^m^CG) in the *Pleurotus* taxa. In light of this, our observed common family expansions of DNMT1/Masc2 in basidiomycetes, including our studied *Pleurotus* taxa, are interesting and warrant further studies.

In higher eukaryotes, besides silencing TEs, DNA methylation also impacts the expression of protein-coding genes that are juxtaposed within or adjacent to TEs^49^. Considering the high density of protein-coding genes in fungal genomes, DNA methylation may play an even greater role due to the closer adjacency of protein-coding genes with their neighboring TEs. Indeed, our results showed that ^m^CG in TEs of the *Pleurotus* taxa are associated with low expression of TEs and their neighboring genes, suggesting a conserved negative regulatory role of DNA methylation on the expression of TEs and their neighboring genes across lower and higher eukaryotes (Fig. 6).

Concerning the independent evolution of Pt and Pe, any discrepancy in the establishment and maintenance of DNA methylation in the respective taxon may result in differentially methylated regions (DMRs). Comparisons of syntenic regions between methylomes of the two taxa have revealed limited DMRs in the genic regions. This is expected given their similar enzyme systems in terms of both composition and sequences in the establishment and maintenance of DNA methylation. Exploration of the potential relationships between these genic DMRs and their morphological and physiological divergence might be a promising approach for uncovering the target genetic loci in *Pleurotus* mushrooms for developing future genetic and epigenetic manipulations to breed better cultivars.

## Materials and Methods

### Strains and culture conditions

Monokaryotic strains of *Pleurotus eryngii* var. *eryngii* (Pe, JKXB130DA) strain and *Pleurotus tuoliensis* (Pt, JKBL130LB) were used for whole genome sequencing, whole genome bisulfite sequencing and RNA sequencing. Both monokaryotic mycelia were cultivated in PDA (Potato Dextrose Agar) liquid medium in the dark at 23 °C for 14 days and stored at −80 °C.

### Genome sequencing and assembly

Genomic DNA of both monokaryotic strains was extracted using the CTAB nuclear preparation method followed by an additional step of phenol-chloroform purification to remove contaminations. Pt and Pe genomes were sequenced using 5 and 6 single-molecule real-time (SMRT) cells, respectively, with P6-C4 chemistry on the PacBio RS II platform (Pacific Bioscience). In total, 554,694 and 609,010 clean reads were generated by SMRT cells for Pt and Pe, respectively. After filtering low-quality raw reads (read quality <0.8), both genomes were assembled using the FALCON-integrate (https://github.com/PacificBiosciences/FALCON-integrate) under the following parameters: initial mapping (length_cutoff = 6000), pre-assembly (length_cutoff_pr = 8000), and overlap filtering setting (–max_diff 100 –max_cov 100 —min_cov 10 —bestn 10) . finisherFC^62^ was used for upgrading the draft assembly and Quiver (SMRT Analysis v2.3.0) was used for genome polishing. Without special notations, all following annotation analyses were carried out for both Pt and Pe genome assemblies.

### Identification and annotation of transposable elements

RepeatModeler v1.0.3 (http://www.repeatmasker.org/RepeatModeler.html) was used to *de novo* identify the transposon elements. LTR-harvest^63^ was used to specifically detect full-length long-terminal repeat (LTR) transposable elements (TEs). Then, output consensus sequences of RepeatModeler and LTR-harvest were merged and clustered with 80% similarity by the CD-hit program^64^ to generate a raw repeat library. After aligning the raw repeat library against the Repbase database^65^ for classification, a newly revised repeat library was constructed and input into RepeatMasker v4.0 (http://www.repeatmasker.org/). Finally, the output of RepeatMasker was parsed by “One code to find them all” script^66^ to obtain the finalized annotation files of transposon elements in both Pt and Pe genomes.

To estimate the insertion time of identified LTR TEs, the Kimura 2-Parameter distance^67^ of paired long-terminal repeats of given full-length LTR TEs was calculated with original Perl scripts. All estimated distances were converted into divergence time in MYA (Million Years Ago) according to the fungal substitution rate of 1.05 × 10^–9^ nucleotides per site per year^68^.

### Gene prediction and annotation

Highly accurate *ab initio* Augustus and GeneMark gene models were predicted by braker v1.9^69^ combined with deep RNA-seq data. Then, the Trinity^70^ and PASA^71^ pipeline was used to generate high-quality gene structures. Additionally, proteomes of *Pleurotus ostreatus*, *Agaricus bisporus*, *Coprinopsis cinerea*, *Laccaria bicolor* and *Fistulina hepatica* downloaded from the JGI Fungi Portal (https://genome.jgi.doe.gov/programs/fungi/index.jsf) were mapped to draft genomes as evidence for proteins. Finally, EVidenceModeler (EVM)^72^ was utilized to compute weighted consensus gene structure annotations based on the foregoing *ab initio* gene models, PASA alignment assemblies, and protein homology data. After the above initial gene prediction, the PASA pipeline was re-utilized to update the EVM consensus predictions by adding UTR annotations. Apollo software^73^ was used to manually check and modify the updated gene models. Finally, BUSCO v1^74^ was used to estimate the integrity of the genomic assembly to align all gene models against the Eukaryota *odb9* dataset (http://busco.ezlab.org/).

Annotations of functional gene models were based on aligning gene sequence to annotated proteins from public databases. Briefly, Blast2GO (https://www.blast2go.com/) was used to classify all genes according to GO (Gene Ontology) terms based on the BLASTP^75^ alignment against the Nr (Non-redundant) database with a threshold e-value <1e-5. InterPro^76^ annotation was also performed by Blast2GO. Proteins of orthologous groups were annotated based on the KOG (euKaryotic Orthologous Groups) database^77^ with the same threshold as GO annotation. KEGG (Kyoto Encyclopedia of Genes and Genomes) annotation was performed by the KAAS (KEGG Automatic Annotation Server) website with the bi-directional hit (BBH) method^78^.

### Phylogenomic analysis

Orthologous gene families in Pt, Pe and other thirteen representative fungi were classified by OrthoMCL^35^ after performing BLASTP with an e-value < 1e-5 and 1,385 single-copy orthologs were detected. For each ortholog group of single-copy genes, multi-sequence alignment of their protein sequences was constructed using MAFFT^79^. Different orthologs of the same species were concatenated into a super sequence alignment involving all orthologous protein sequences across all sampled taxa. Gblocks 0.91b^80^ was used to remove un-conserved alignments in the foregoing super sequence alignment. The phylogenomic tree was conducted by FastTree^81^ with the model of LG+CAT based on the super sequence alignment, which was validated by the bootstrap method (1,000 bootstrap values). According to several fossil calibration points of Agaricales (AGA), Boletales (BOL) and Ascomycota (ASC)^37^, r8s^82^ was performed to estimate the divergent time nodes among the three *Pleurotus* species by the TN algorithm and PL methods.

### Characterization of genes encoding key featured enzymes

Profiles of important wood-decay-related gene families, DNA methyltransferases, and enzymes involved in siRNA biogenesis and silencing were characterized by essential domain searching in related databases. Specifically, putative genes encoding CAZymes (Carbohydrate-Active Enzymes) were identified by running an annotation pipeline in the dbCAN database^83^ based on the criterion of e-value < 1e-17 and coverage > 0.45. HMMER (http:// hmmer.org/) was used for searching cytochrome P450 candidate genes, which were aligned to the fungi P450 database^84^ for further filtering and naming. Other decay-related gene families were annotated with HMMER by searching for corresponding protein domains from the Pfam database v31.0 (http://pfam.xfam.org/) (Table S11) in whole proteomes with an e-value cutoff of 1e-5.

Enzymes involved in epigenetic modifications, including DNA methyltransferase (PF00145, C-5 cytosine-specific DNA mythyltransferase domain), RdRP (RNA dependent RNA Polymerase; PF05183, RNA dependent RNA polymerase domain), DCL (Dicer-like proteins; PF00636, Ribonuclease III domain) and Argonaute proteins (PF02171, Piwi domain) were also annotated by a similar domain searching method using the domains parenthesized and tabulated as above. MEGA6.0^85^ was used to construct an ML (Maximum Likelihood) phylogenetic tree with the WAG model based on the alignments of domain sequences completed by MAFFT.

### Synteny analysis

To obtain synteny blocks based on the similarities of genomic sequences, the nucmer module of MUMmer3.0^86^ was used to complete pairwise genomic alignments among Po, Pt and Pe. The respective synteny blocks were identified by DAGchainer^87^ with the criteria of harboring cutoff distances less than 20 kb apart and at least 5 sequence pairs per synteny block. The raw synteny blocks were further filtered using QUOTA-ALIGN^88^ to leave one-by-one blocks and exclude blocks related with ancient duplication events. Similarly, to obtain synteny blocks based on the similarities of protein sequences, protein sequences from Po, Pt, and Pe were pairwise aligned by BLASTP and synteny blocks were identified with a cutoff distance of 10 genes apart and at least 5 gene pairs per synteny block. Circos (http://circos.ca/) was used to confirm and visualize the synteny relationship among the three *Pleurotus* genomes.

### Methyl C-seq data processing

Intact genomic DNA extracted from monokaryotic mycelia of Pt and Pe were used for whole-genome bisulfite sequencing (Methyl C-seq) on the Illumina Hiseq 2500 platform (Illumina; San Diego, USA) with standard protocols. After removing low-quality reads using Trimmomatic^89^, the cleaned reads were aligned to each draft genome by Bismark^90^ with 1-bp mismatch, and only uniquely mapped reads were preserved. Moreover, cytosine sites with ≥5 unique mapped reads were taken into account for downstream analysis.

For DMRs (Differentially Methylated Regions) analysis, synteny blocks (identified by genomic sequences) between Pt and Pe were aligned and split into continuous 200 bp regions with step sizes of 100 bp. Then, DMRs between Pt and Pe were discriminated by comparing methylation levels within each 200 bp region by a binomial test and following FDR correction (q-value < 0.05). Finally, overlapped 200 bp DMRs were merged according to the same DMR characteristics (hyper- or hypo-methylated).

### mRNA sequencing data processing

mRNA extracted from monokaryotic mycelia of both Pt and Pe were used for mRNA sequencing on the Illumina Hiseq 2500 platform (Illumina; San Diego, USA) with a standard protocol. After removing low-quality reads using Trimmomatic^89^, the cleaned reads were aligned to each draft genome by Hisat2 with 1-bp mismatch and the expression level (FPKM, Fragment per Kilobase of transcript per Million mapped reads) of each gene model were calculated by Stringtie^91^.

### Small RNA sequencing data processing

Small RNA libraries of monokaryotic mycelia for both Pt and Pe were prepared according to the standard workflow of Illumina TrueSeq Small RNA Preparation Kits and were sequenced on the Illumina Hiseq 2500 platform (Illumina; San Diego, USA). Initially, adaptor contamination of raw reads was removed by cutadapt (http://cutadapt.readthedocs.io/en/stable/). Moreover, noisy reads derived from tRNA, rRNA and snRNA were determined by aligning adaptor-removed raw reads to GtRNAdb (http://gtrnadb.ucsc.edu/), SILVA (https://www.arb-silva.de/), Pfam and snoPY (http://snoopy.med.miyazaki-u.ac.jp/) databases, which were removed ahead of microRNA (miRNA) prediction. miRNA prediction was completed using miRDeep^92^ following a previously published method^93^. siRNA reads were obtained by removing all possible miRNA reads and were further mapped to draft genomes by Bowtie^94^ with 1-bp mismatch. Adjacent siRNAs located within distances less than 100 bp were assigned to the same raw cluster. Raw siRNA clusters with an abundance of at least 5 RPM (Reads per Million reads) and 10 different reads were accepted as the final siRNA clusters.

### Statistics

All Statistical tests in this paper were performed using basic packages in R language (Version 3.3.1, https://www.r-project.org/).

### Accession codes

The Whole-Genome Shotgun project for *P. eryngii* var. *eryngii* and *P. tuoliensis* has been deposited in the NCBI BioProject under PRJNA450170 and PRJNA450171, respectively. Sequencing reads of transcriptome, methylome and small RNA have been deposited in the Sequence Read Archive (SRA) database SRP139982.

## Electronic supplementary material

Supplementary Information

Supplementary Dataset
