# Supplementary material for: The evolution of genomic and epigenomic features in two Pleurotus fungi
Source: Sci Rep. 2018 May 29;8:8313. doi: 10.1038/s41598-018-26619-7 (PMC5974365; doi:10.1038/s41598-018-26619-7)
Supplement: Supplementary file 1 — Supplementary Information [file 41598_2018_26619_MOESM1_ESM.docx]

**The evolution of genomic and epigenomic features in two *Pleurotus* fungi**

Zhibin Zhang^1^**^†^**, Jiawei Wen^1,2^**^†^**, Juzuo Li^1^, Xintong Ma^1^, Yanan Yu^1^, Xiao Tan^2^, Qi Wang^3^, Bao Liu^1^, Xiaomeng Li^1^, Yu Li^3^* and Lei Gong^1^*

**Supplemental Informations**

**Table S1. The comparison of genomic information between *Pleurotus tuoliensis* of strain 489P1 and Pt as well as among *Pleurotus eryngii* of ATCC 90797, strain 183 and Pe.**

|  | 489P1* | Pt | ATCC 90797** | strain 183*** | Pe |
| --- | --- | --- | --- | --- | --- |
| Number of contigs/scaffolds | 500 | 106 | 609 | 199 | 153 |
| Length of the genome assembly (Mb) | 40.8 | 48.2 | 44.6 | 43.8 | 49.9 |
| Contig/Scaffold N50 (Mb) | 0.63 | 1.08 | 0.24 | 0.39 | 0.55 |
| GC content (%) | 50.2 | 50.01 | 48.04 | 49.30 | 49.07 |
| Number of protein-coding genes | 14263 | 13097 | 15960 | 14443 | 13213 |
| Average gene length (bp) | - | 1717 | 1621 | - | 1607 |
| Average coding sequence size (bp) | 1308 | 1186 | 1318 | - | 1069 |
| Average number of exons per gene | - | 6.4 | 5.6 | - | 6.4 |
| Average exon size (bp) | - | 205 | 236 | - | 185 |
| Average intron size (bp) | - | 73 | 68 | - | 79 |
| Average size of intergenic regions (bp) | - | 1932 | 1174 | - | 2145 |
| TE content (%) | 3.43 | 20.0 | - | - | 19.4 |

* Assembly was derived from Guo et al. (ref 18 in manuscript).

** Assembly was derived from JGI Fungi Portal (<https://genome.jgi.doe.gov/programs>

/fungi/index.jsf).

*** Assembly was derived from Yang et al. (ref 19 in manuscript).

**Table S2. Gene models of *Pleurotus tuoliensis* (Pt) and *P. eryngii* var*. eryngii* (Pe) supported by public databases.**

|  | Pt | Pe |
| --- | --- | --- |
| Nr | 12119 | 12344 |
| GO | 7066 | 7206 |
| KEGG | 3243 | 3027 |
| Interpro | 10094 | 10054 |
| KOG | 5281 | 5323 |
| Total | 13097 | 13213 |

**Table S3.** **Summary of annotation of repeat sequences in Pt, Pe and *Pleurotus ostreatus* (Po)*.***

|  | Number of elements | Length (bp) | Percentage (%) |
| --- | --- | --- | --- |
| Pt: |  |  |  |
| LTR/gypsy | 2002 | 4745001 | 9.84 |
| LTR/copia | 360 | 801306 | 1.66 |
| Other-LTR | 59 | 118059 | 0.24 |
| LINE | 112 | 279420 | 0.58 |
| DNA | 64 | 266697 | 0.55 |
| unknown | 4928 | 3419811 | 7.10 |
| Pe:  LTR/gypsy | 2717 | 5095694 | 10.21 |
| LTR/copia | 410 | 900229 | 1.80 |
| Other-LTR | 43 | 71920 | 0.14 |
| LINE | 77 | 171178 | 0.34 |
| DNA | 91 | 359966 | 0.72 |
| unknown | 4945 | 3062891 | 6.14 |
| Po: |  |  |  |
| LTR/gypsy | 792 | 1707000 | 4.97 |
| LTR/copia | 145 | 232200 | 0.68 |
| Other-LTR | 35 | 36900 | 0.11 |
| LINE | 40 | 46700 | 0.14 |
| DNA | 39 | 96600 | 0.28 |
| unknown | - | 1236300 | 3.6 |

**Table S4. Evaluation of genome completeness using BUSCO according 303 eukaryota orthologous genes for Pt and Pe.**

|  | Complete (%) | Fragmented (%) | Missing (%) |
| --- | --- | --- | --- |
| Pt | 89.8 | 6.9 | 3.3 |
| Pe | 73.6 | 18.8 | 7.6 |

**Table S5.** **Summary of pairwise syntenic blocks and homologous gene numbers among Pt, Pe and Po.**

|  | Blocks | Gene numbers |
| --- | --- | --- |
| Pt vs. Po | 81 | 8407(64.2%) vs. 8159(66.2%) |
| Pe vs. Po | 111 | 8112(61.4%) vs. 7705(62.5%) |
| Pt vs. Pe | 132 | 7997(61.1%) vs. 8146(61.7%) |

*****The contents of Table S6 through S9 are enclosed in the excel sheets*****

**Table S5.** **Top 10 abundant gene ontogoly (GO) categories of genes that are common conserved in three *Pleurotus* genomes and specific in Pt or Pe.**

**Table S6.** **Numbers of carbohydrate-active enzymes identified in 15 fungi genomes according to the CAZy database.**

**Table S7.** **Gene IDs of 10 oxidoreductase and 16 CAZyme families in 15 fungi genomes.**

**Table S8.** **Top 10 abundant gene ontogoly(GO) categaries of genes that are enclosed in identified DMRs in Pt and Pe at their stage of mycelium.**

**Table S10. Number of three key classes enzymes that correlate with siRNA biogenesis and silencing in 15 fungi genomes.**

| Species | RdRP | Argonaute | DCL |
| --- | --- | --- | --- |
| *Agaricus bisporus* | 6 | 6 | 3 |
| *Coniophora puteana* | 7 | 6 | 3 |
| *Coprinopsis cinerea* | 8 | 8 | 3 |
| *Cryptococcus neoformans* | 1 | 1 | 2 |
| *Dichomitus squalens* | 9 | 7 | 3 |
| *Fistulina hepatica* | 1 | 5 | 3 |
| *Hydnomerulius pinastri* | 5 | 9 | 3 |
| *Laccaria bicolor* | 6 | 6 | 3 |
| *Neurospora crassa* | 2 | 4 | 2 |
| *Phlebiopsis gigantea* | 7 | 12 | 3 |
| *Pisolithus microcarpus* | 5 | 8 | 3 |
| *Pleurotus eryngii* var*. eryngii* | 5 | 7 | 4 |
| *Pleurotus ostreatus* | 5 | 7 | 4 |
| *Pleutotus tuoliensis* | 7 | 7 | 4 |
| *Tremella mesenterica* | 2 | 2 | 1 |

**Table S11. Pfam IDs of 10 oxidoreductases for HMMER search**

| Genes | Pfam ID |
| --- | --- |
| POD | PF00141,PF11895 |
| MCO | PF00394, PF07731,PF07732 |
| CRO | PF01822, PF07250,PF09118 |
| CDH | PF16010,PF00732,PF05199 |
| Cytb | PF16010,PF00734 |
| OXO | PF00190,PF07883 |
| QRD | PF03358,PF02525 |
| DyP | PF04261 |
| HTP | PF01328 |
| P450 | PF00067 |

**
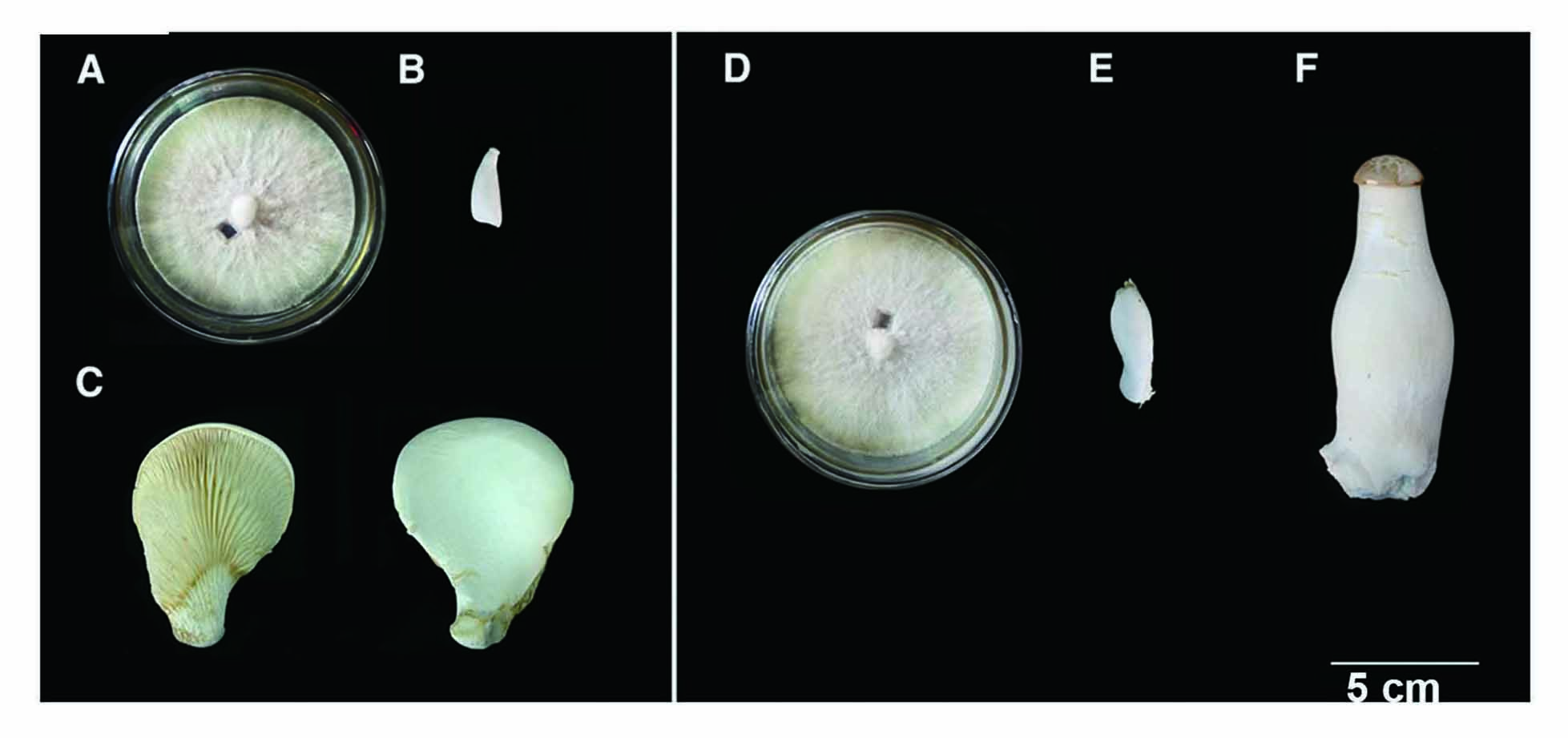
**

**Figure S1. Phenotypic illustrations of *Pleurotus tuoliensis* (Pt) and *P. eryngii* var*. eryngii* (Pe)**. (A) through (C) and (D) through (F) illustrate the status of Pt and Pe at development stages of monokaryotic mycelia, primordial and fruit body, respectively.


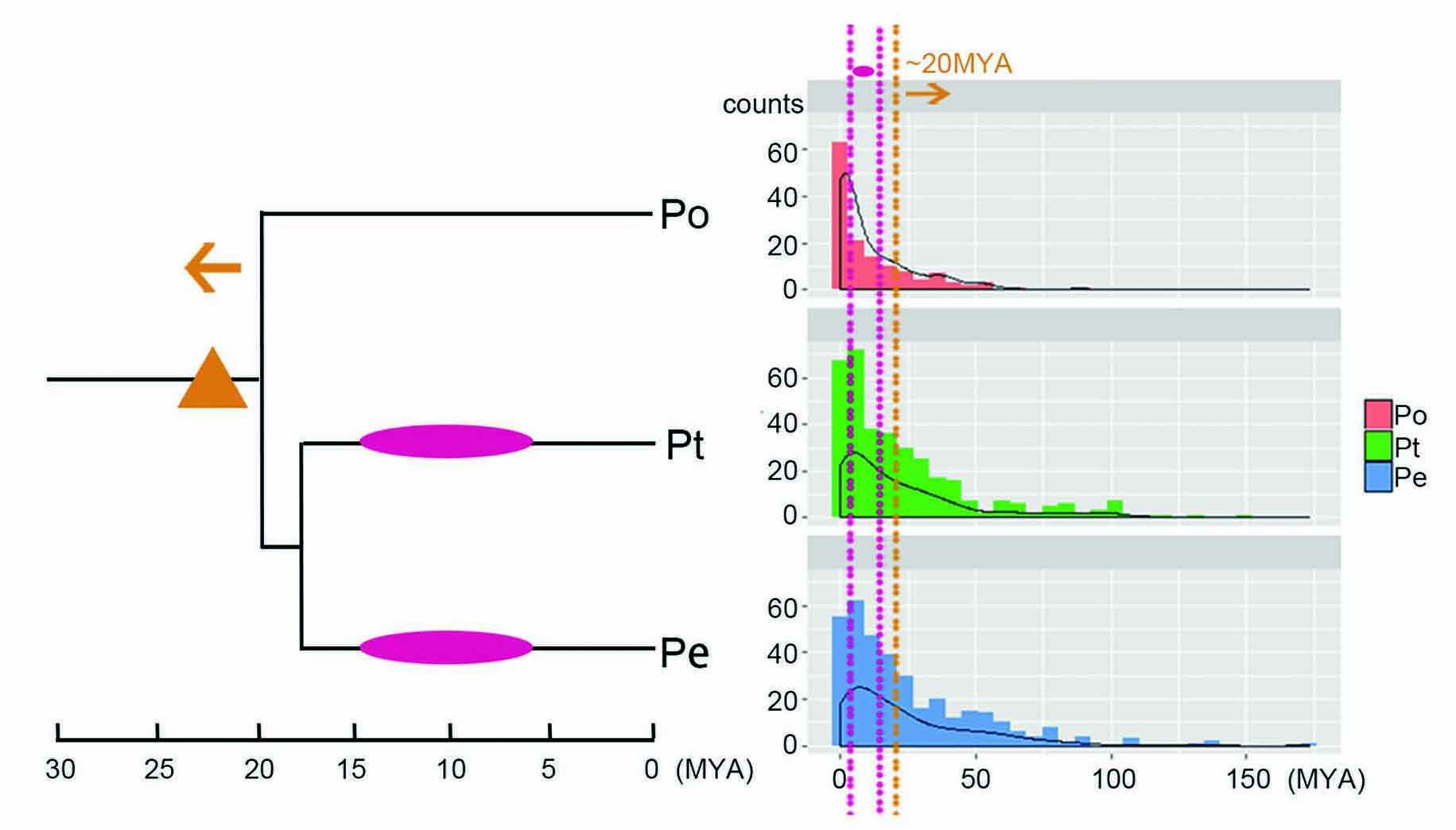


**Figure S2. Estimation of insertion time of LTR retro-transposons in *Pleurotus ostreatus* (Po), Pt, and Pe.** Left panel: the estimated insertion/burst time ranges in Pt and Pe are denoted as the pink ovals on the phylogenetic tree; the estimated divergence time-point of Po (~20 MYA) from common ancestor of Pt and Pe is labeled in brown triangle. Right panel: the histogram and density curves of estimated LTR retro-transposon insertion/burst time in Po, Pt, and Pe. Similar pink dashed lines flanking ovals denotes similar estimated insertion/burst time ranges of LTR TEs in Pt and Pe. Brown dashed lines with arrows pointing towards those ancient insertion/burst of LTR TEs occurred before divergence of Po from common ancestor of Pt and Pe.

**
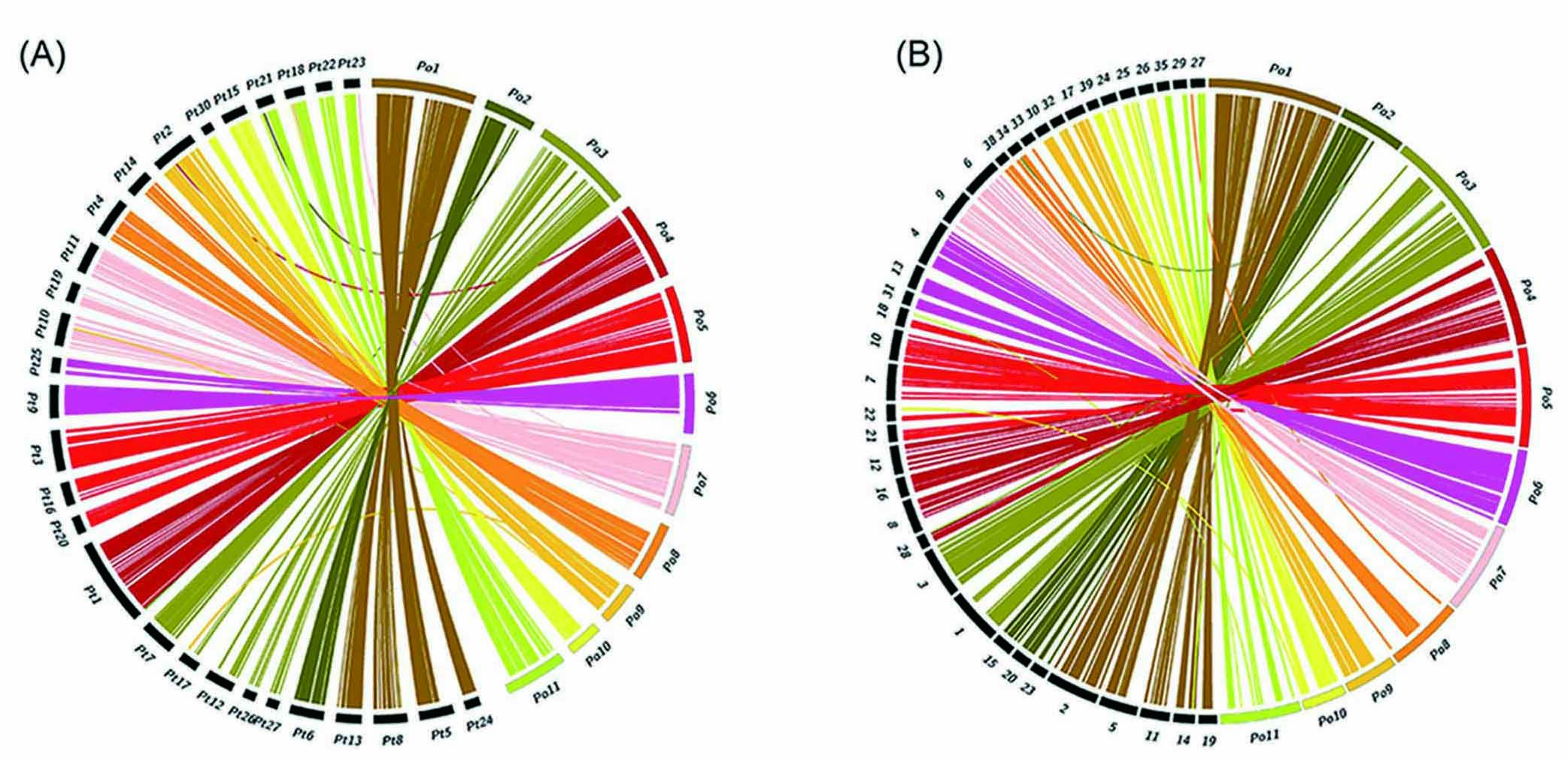
**

**Figure S3. Syntenic regions in Pt, Pe and Po.** The syntenic correspondence Po vs. Pt and Po vs. Pe at nucleic acid level are depicted in panel A and B, respectively. colorful stripes represent chromosomes derived from Po and black stripes represent contigs from Pt (A) and Pe (B), respectively.

**
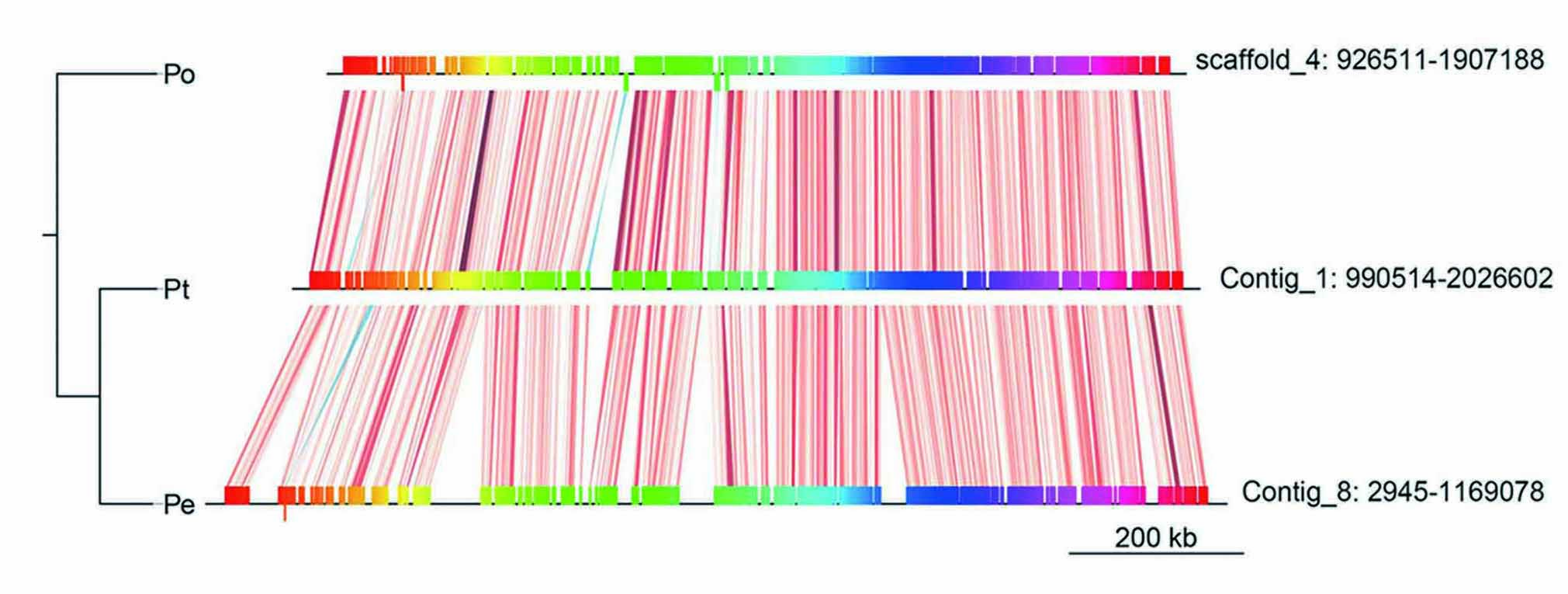
**

**Figure S4. Exemplary syntenic blocks among gene homologs of Po, Pt, Pe.** Colorful blocks represent genes located on the corresponding contig or chromosome and linked lines represent the homologous relationships between the gene pairs referred by their protein similarities. Genes under the contig or chromosome lines indicate they are conversely paired with gene homologs on the other contig or chromosome.

**
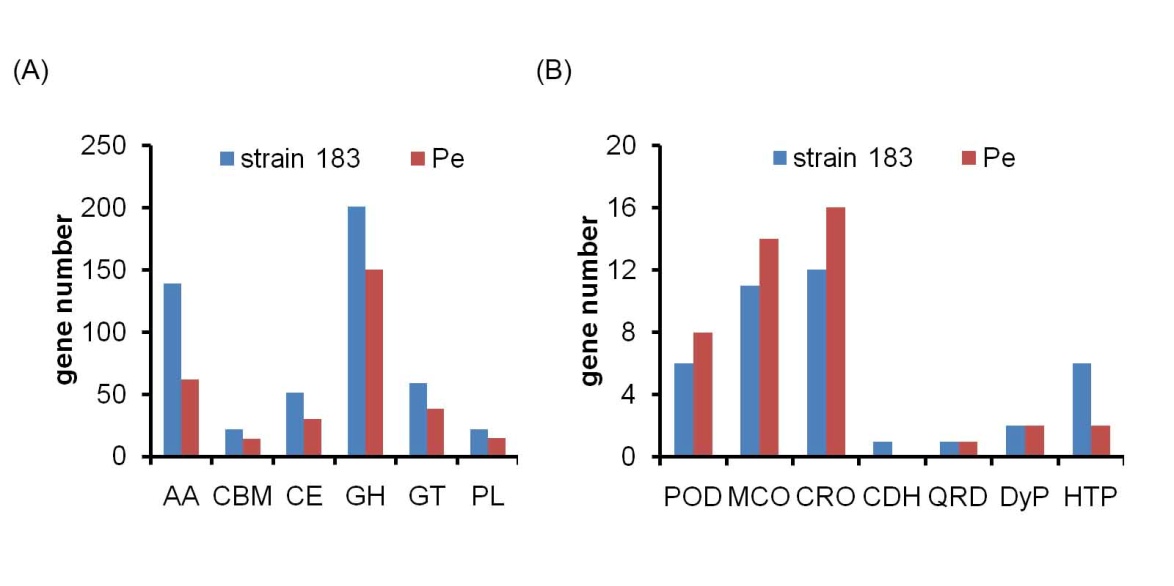
**

**Figure S5.** **Number of CAZyme-coding genes (A) and important oxido-reductase genes (B) in both assemblies of strain 183 and Pe, respectively.** Abbreviations of gene family names: AA, Auxiliary Activities; CBM, Carbohydrate-Binding Modules; CE, Carbohydrate Esterases; GH, Glycoside Hydrolases; GT, GlycosylTransferases; PL, Polysaccharide Lyases; POD, class II peroxidases; MCO, multicopper oxidases; CRO, copper-radical oxidases; CDH, cellobiose dehydrogenase; QRD, quinone reductases; DyP, dye-decolorizing peroxidases; HTP, heme-thiolate peroxidases.


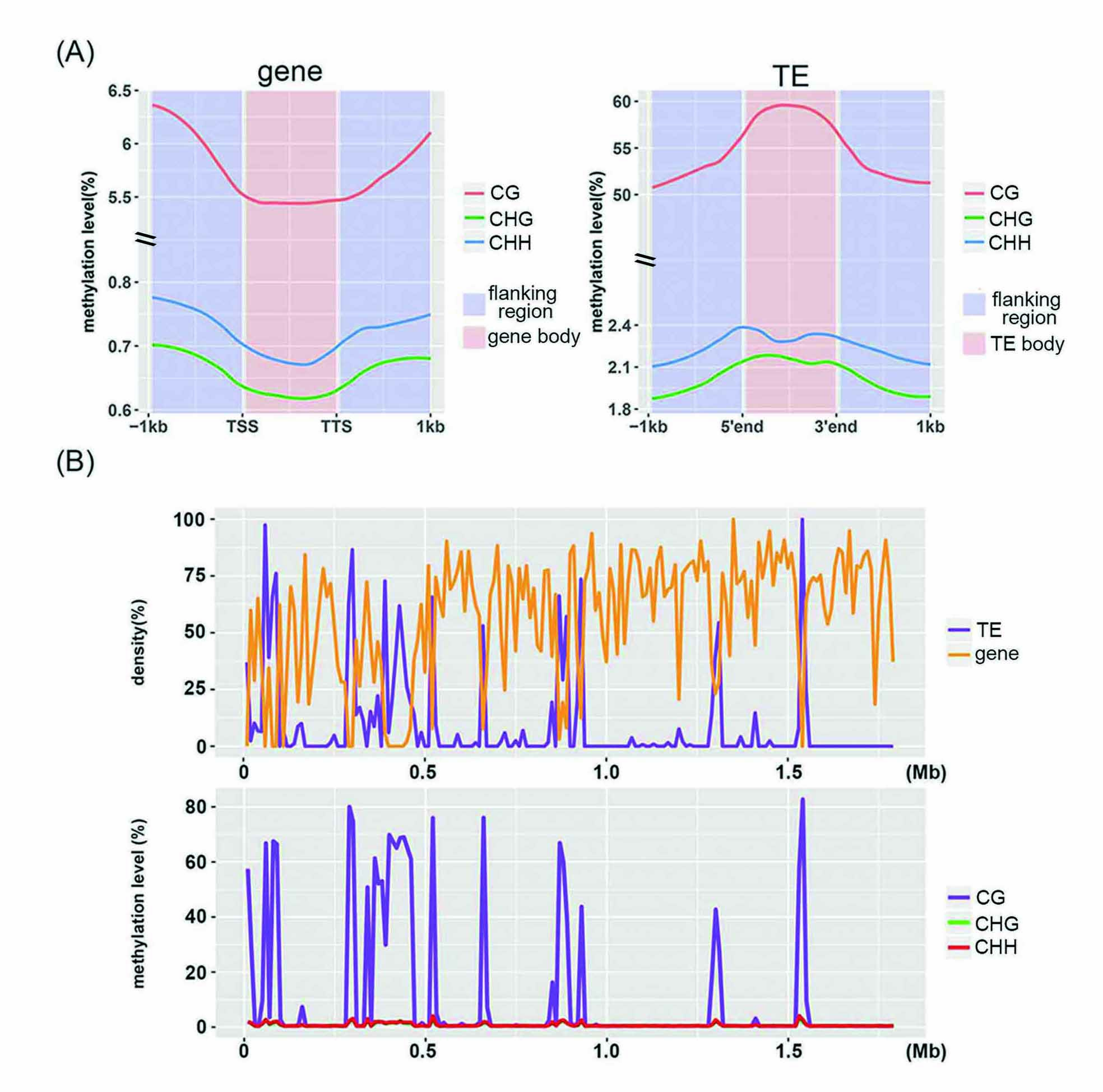


**Figure S6. DNA methylation profiles in Pe.** (A) Distribution of DNA methylation levels (in the contexts of CG, CHG and CHH, bottom) and density curves of genes and TEs along the contig 1 of Pe genome; (B) Averaged DNA methylation levels within (enclosed between TSS and TTS) and around (flanking +1kb) genic and TE regions.

**
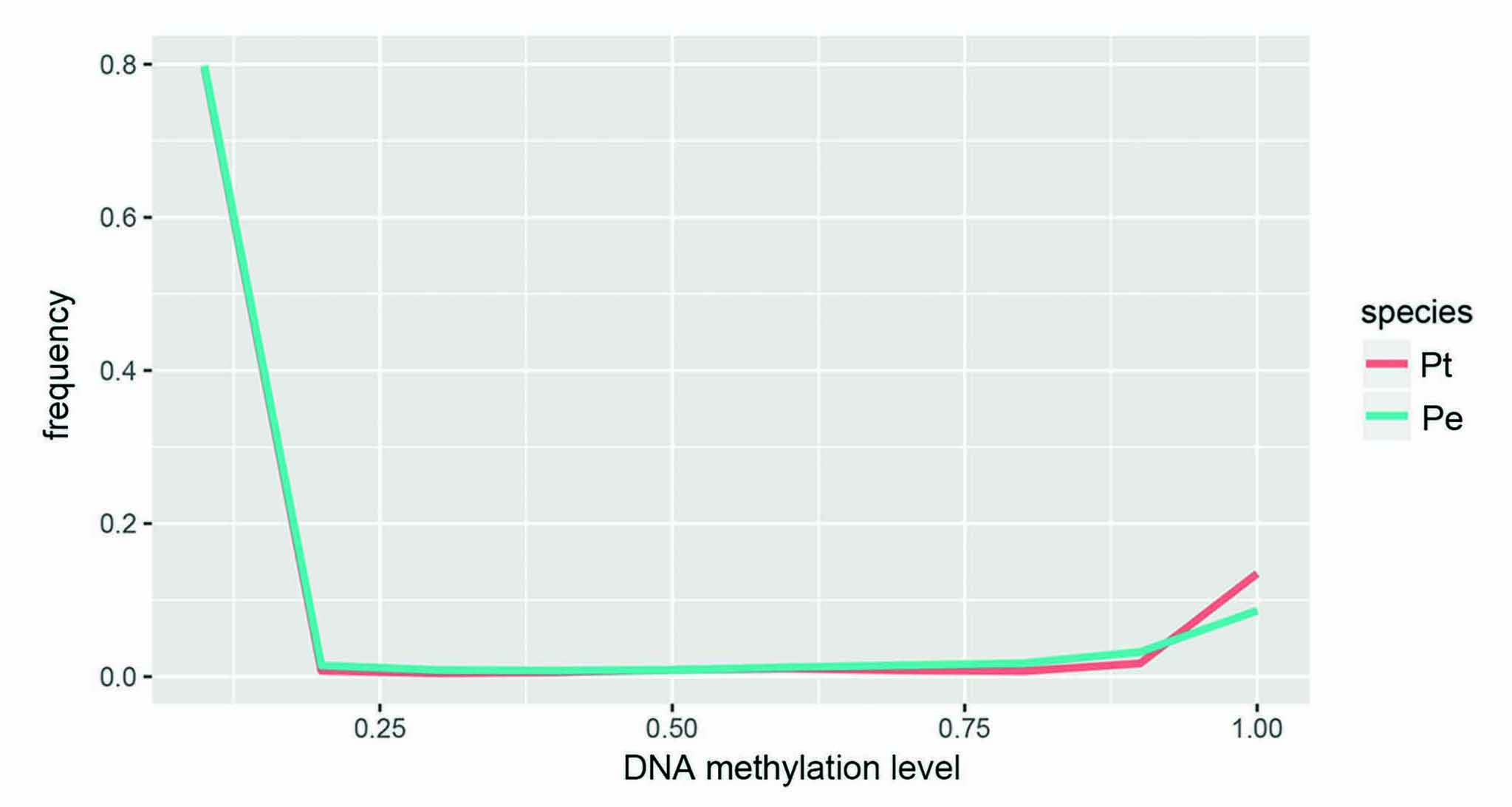
**

**Figure S7. The line chart of DNA methylation levels in CG context in Pt and Pe.** Both plots show the bimodal distribution of DNA methylation in CG context.


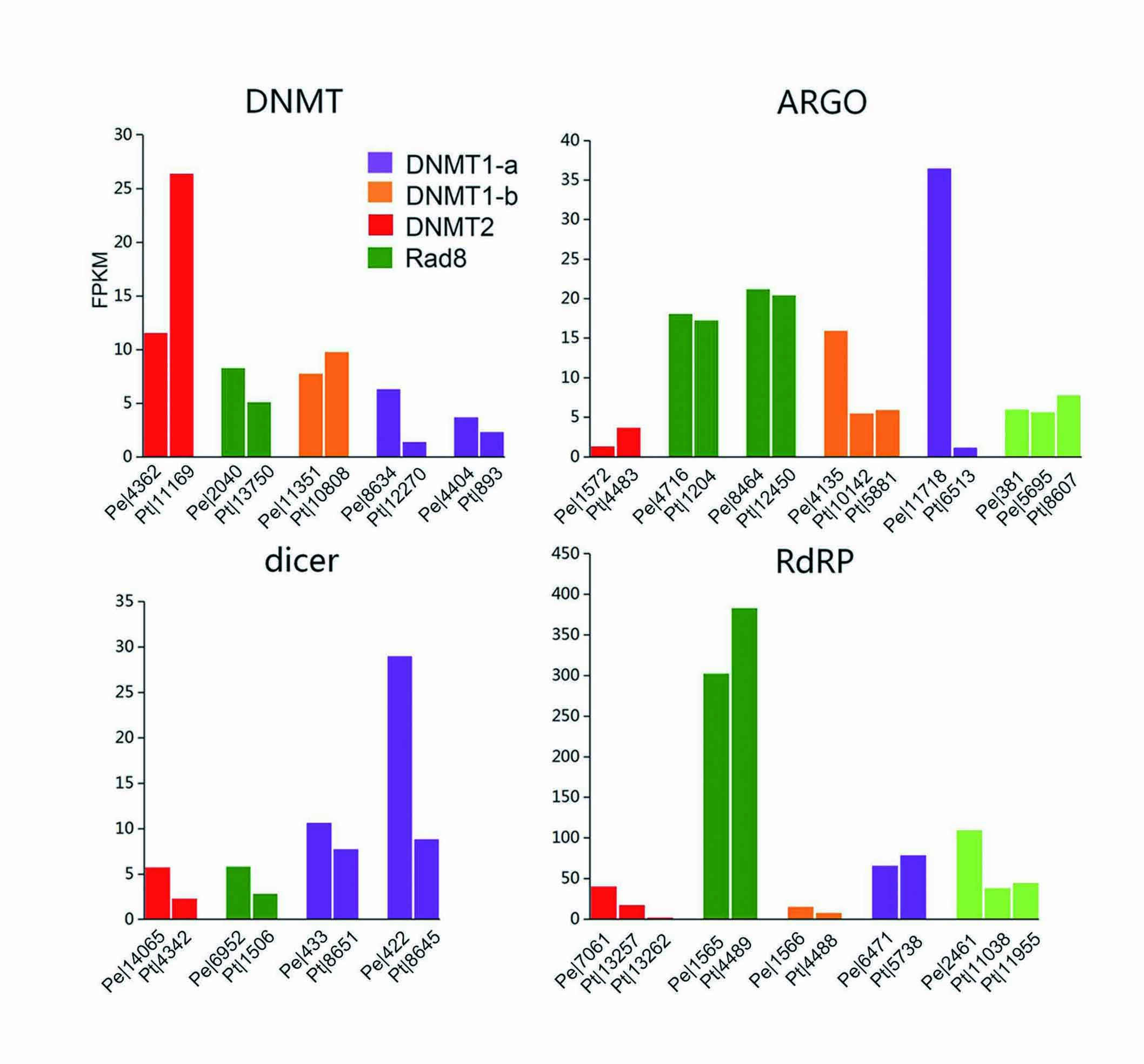


**Figure S8. Expression levels of genes that encode DNA methyltransferase and correlate with siRNA biogenesis and silencing in Pt and Pe.** Genes in the same color represent they belong to the same group of gene homologs.

**
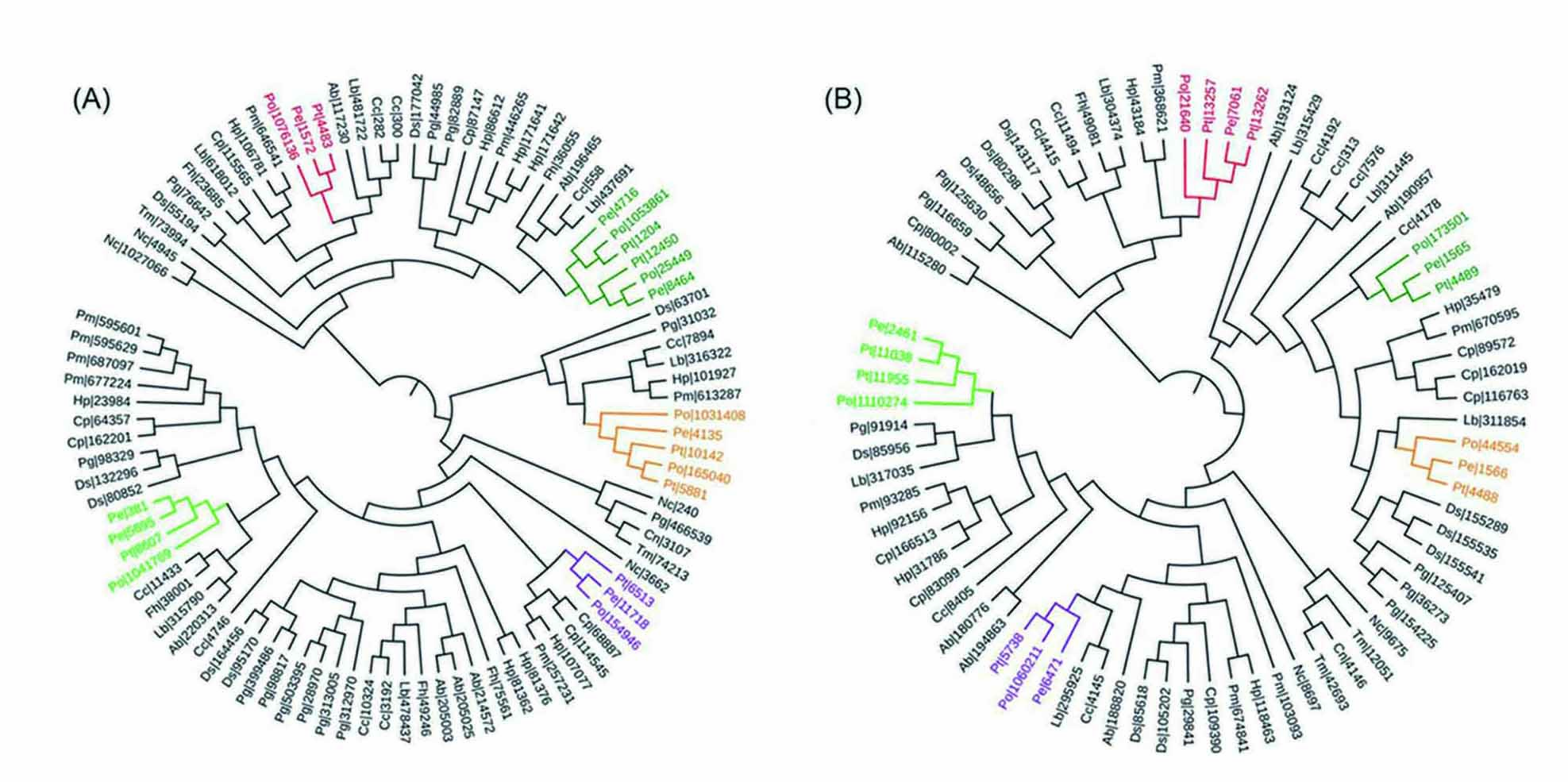
**

**Figure S9. Phylogenetic tree of gene homologs encoding RdRP and ARGO in fifteen fungi species.** The Maximum Likelihood (ML) phylogenetic trees of genes encoding (A) RdRP and (B) Argonaute were constructed as described in Figure 4 based on Ribonuclease III domain (PF00636) and Piwi domain (PF02171), respectively, and different colors denotes diverged *Pleurotus* RdRP and Argonaute genes, respectively.


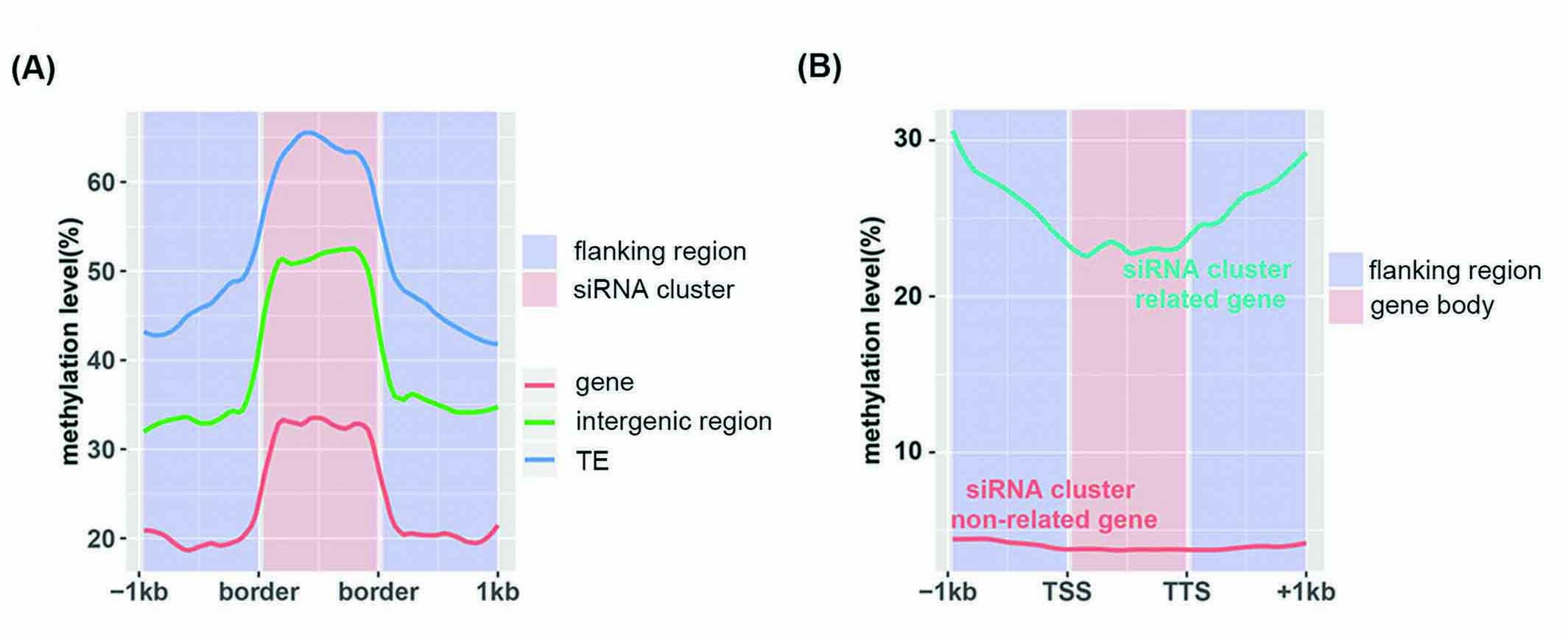


**Figure S10. Establishment of DNA methylation profiles in Pe.** (A) DNA methylation level of genes, TEs, and other types of regions within and around regions with siRNA clusters in Pe; (B) DNA methylation level within and around genes that are related and non-related with siRNA clusters in Pe (Materials and Methods).


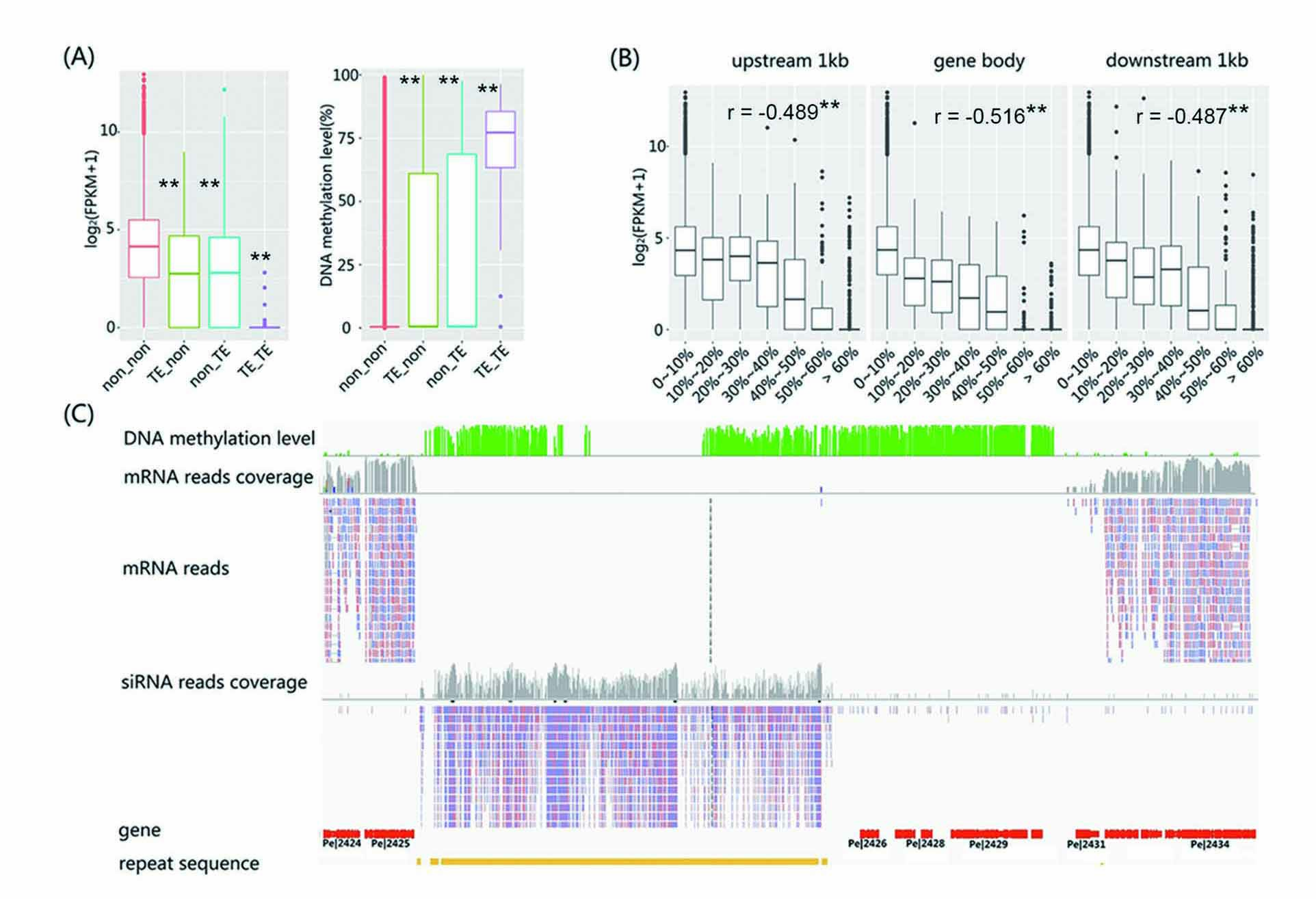


**Figure S11. DNA methylation around TE regions negatively regulate expression of adjacent genes in Pe.** (A) Negative impacts of DNA methylation in/around TEs (on the right) on the expression of their neighboring genes (evaluated as FPKM values, on the left) in Pe. “TE_TE”, “non_TE”, “TE_non”, and “non_non” denotes genes flanked with TEs on both upstream and downstream sides, only flanked with downstream TEs, only flanked with upstream TEs, and without any flanking TEs, respectively; (B) Significant negative correlation between gene expression and DNA methylation level within corresponding genic regions. On the x axis, genes are categorized into bins of hierarchical DNA methylation levels; (C) Exemplary overviewed IGV tracks illustrate the relative distributions of DNA methylation level, mRNA reads abundance, siRNA reads abundance along the ~52 kb region in contig 14:102,531-154492 of Pe.
